# Identification of a Novel Small RNA Encoded in the Mouse Urokinase Receptor uPAR Gene (*Plaur*) and Its Molecular Target *Mef2d*

**DOI:** 10.3389/fnmol.2022.865858

**Published:** 2022-07-06

**Authors:** Karina D. Rysenkova, Konstantin E. Troyanovskiy, Polina S. Klimovich, Taisiya R. Bulyakova, Ekaterina M. Shelomentseva, Anna A. Shmakova, Daria Yu. Tanygina, Olga I. Ivashkina, Konstantin V. Anokhin, Maxim N. Karagyaur, Maria I. Zvereva, Kseniya A. Rubina, Vsevolod A. Tkachuk, Ekaterina V. Semina

**Affiliations:** ^1^Institute of Experimental Cardiology, National Medical Research Centre of Cardiology named after academician E.I. Chazov, Moscow, Russia; ^2^Faculty of Medicine, Lomonosov Moscow State University, Moscow, Russia; ^3^Institute for Advanced Brain Studies, Lomonosov Moscow State University, Moscow, Russia; ^4^Laboratory of Neurobiology of Memory, P.K. Anokhin Research Institute of Normal Physiology, Moscow, Russia; ^5^Laboratory of Neuroscience, National Research Center “Kurchatov Institute”, Moscow, Russia; ^6^Department of Chemistry, Lomonosov Moscow State University, Moscow, Russia

**Keywords:** urokinase receptor, uPAR, *Plaur*, *Plaur*-miR1-5p, *Mef2d*, neuroblastoma, *Plaur*-miR1-3p, Neuro2A

## Abstract

Urokinase receptor (uPAR) is a glycosylphosphatidylinositol (GPI)-anchored receptor of urokinase (uPA), which is involved in brain development, nerve regeneration, wound healing and tissue remodeling. We have recently shown that *Plaur*, which encodes uPAR, is an early response gene in murine brain. Assumingly, diverse functions of *Plaur* might be attributed to hypothetical, unidentified microRNAs encoded within introns of the *Plaur* gene. Using a bioinformatic approach we identified novel small RNAs within the *Plaur* gene and named them Plaur-miR1-3p and Plaur-miR1-5p. We confirmed *Plaur*-dependent expression of Plaur-miR1-3p and Plaur-miR1-5p in the mouse brain and mouse neuroblastoma Neuro2a cells. Utilizing an *in silico MR-microT* algorithm in DianaTools we selected two target genes – *Mef2d* and *Emx2* with the highest binding scores to small RNAs selected from identified Plaur-Pre-miR1. Furthermore, sequencing of mouse brain samples for Plaur-miR1-5p target genes revealed two more genes—*Nrip3* and *Snrnp200*. The expression of *Emx2, Mef2d*, and *Snrnp200* in the mouse brain and *Mef2d* and *Snrnp200* in Neuro2a cells correlated with expression of *Plaur* and small RNAs—Plaur-miR1-3p and Plaur-miR1-5p. Finally, we demonstrated elevated MEF2D protein expression in the mouse brain after *Plaur* induction and displayed activating effects of Plaur-miR1-5p on *Mef2d* expression in Neuro2a cells using Luciferase reporter assay. In conclusion, we have identified Plaur-miR1-3p and Plaur-miR1-5p as novel small RNAs encoded in the *Plaur* gene. This finding expands the current understanding of *Plaur* function in brain development and functioning.

## Introduction

Urokinase receptor (uPAR, also known as CD87, U-PAR and UPAR; encoded by the gene *Plaur*) is a multifaceted protein with numerous physiological and pathological effects. Since uPAR is anchored to the plasma membrane via glycosylphosphatidylinositol (GPI)-moiety, it can move laterally within the membrane leaflet, forming the uPAR interactome. The final outcome and cellular effects of uPAR-mediated interactions depend on the sum of signals coming from the transmembrane partners and receptors engaged in uPAR interactome (Eden et al., 2011). Urokinase (uPA) binding to uPAR activates the uPA/uPAR complex at the leading edge of migrating cell promoting their migration via extracellular matrix remodeling. These changes contribute to various physiological processes—morphogenesis, tissue regeneration and nerve fiber growth (Parfenova et al., 2009; Tkachuk et al., 2009; Semina et al., 2016; Klimovich et al., 2020; Yepes et al., 2021), as well as to pathophysiological processes—fibrosis, tumor growth and metastasis (Mahmood et al., 2018; Tkachuk et al., 2019).

uPAR overexpression stimulates radial neuronal migration to the outer layers of differentiating cortex (Shmakova et al., 2021), whereas uPAR knockout reduces migration of parvalbumin-expressing GABA interneurons into cerebral cortex (Powell et al., 2003). Recent papers have shown that mutations and polymorphisms in the *Plaur* gene or uPAR ligand SRPX2 affect the formation of brain structures and induce severe developmental pathologies in humans (speech deficiency, mental weakness and autism spectrum disorders) (Bruneau and Szepetowski, 2011). Using a model of acute generalized seizures in mice, we revealed that *Plaur* operates as an immediate early gene, and is rapidly induced by neuronal activity in different brain regions independently of *de novo* protein synthesis (Shmakova et al., 2020). This rapid and universal response confirms an important role of uPAR in neuronal response to excitation and/or damage.

We have previously demonstrated that CRISPR/Cas9-mediated targeting of the *Plaur* gene inhibits Neuro2a neuroblastoma cell proliferation, leading to downregulation of full-length *Ntrk3* messenger RNA (mRNA), which encodes tropomyosin receptor kinase C (TrkC), a receptor that is involved in p38/Akt signaling pathway (Rysenkova et al., 2018). However, the reported effect of *Plaur* knockout on *Ntrk3* mRNA expression may not be merely attributed to uPAR-dependent interactome function. It has been previously established that gene expression and mRNA functioning can be regulated by so-called non-coding RNAs that are not translated into a protein. Being a part of this group, microRNAs (miRNAs) represent a highly conserved fraction of short RNA (18–27 nt) endogenously produced in many organisms. A complex secondary structure of miRNA precursor (pri-miRNA) is subsequently processed into a more mature form of pre-miRNA with a hairpin structure, which is further transformed into a mature form located at the 5′ or 3′ end of the loop (Broughton et al., 2016). According to the canonical pathway, mature miRNAs complementarily interact with their target transcripts in mammalian cells, leading to mRNA degradation or translation inhibition (Broughton et al., 2016).

Originally identified in the cytoplasm, miRNAs have now been found in all cellular compartments, where their functions are not limited to target mRNA degradation. Indeed, there is evidence indicating that miRNAs could either suppress or activate gene expression by engaging with target gene promoters in the nucleus (Broughton et al., 2016). Currently, over 2,600 human miRNAs have been registered in the miRBase database,^1^ which correspond to 2,500 mouse miRNAs owing to their high conservatism (Ørom et al., 2008; Place et al., 2008). The miRBase database (see text footnote 1 Release 22.1: October, 2018) comprises about 2,654 mature human miRNAs in contrast to only 1,978 mature mouse miRNAs [GRCm38]).

The *Plaur* gene consists of seven exons and six introns. Prior to our study, no miRNAs have been reported in the *Plaur* gene sequence. The total *Plaur* gene size is 16,000 bp, while the mature mRNA (merely exons) is composed of only 1,000 nt (Kjaergaard et al., 2008), suggesting that non-coding RNAs, including miRNAs, could be encoded in this gene. We conducted a bioinformatic search and analyzed the miRNAs that are encoded in the *Plaur* gene. We identified novel small RNAs and named them Plaur-miR1-3p and Plaur-miR1-5p. Using wild-type uPAR-expressing Neuro2a cells, CRISPR-edited uPAR-deficient Neuro2a cells and *in vivo* model of endogenous induction of *Plaur* expression in the brain, we confirmed the existence of these new small RNAs, Plaur-miR1-3p and Plaur-miR1-5p. *In silico* analysis of target genes allowed us to identify its possible functions, namely determination of the cell fate and a crucial role in neuronal apoptosis in the developing central nervous system. We confirmed *Plaur*-dependent expression of Plaur-miR1-3p and Plaur-miR1-5p in the mouse brain and mouse neuroblastoma Neuro2a cells. Utilizing an *in silico* MR-microT algorithm in DianaTools we selected two target genes—*Emx2* and *Mef2d*—with the highest binding score. Moreover, sequencing of the mouse brain samples for Plaur-miR1-5p target genes revealed two more targets—*Nrip3* and *Snrnp200*. The expression of *Emx2*, *Mef2d* and *Snrnp200* in the mouse brain and *Mef2d* and *Snrnp200* in Neuro2a cells correlated with expression of the *Plaur* gene and small RNAs—Plaur-miR1-3p and Plaur-miR1-5p. Finally, in the mouse brain we demonstrated an elevated expression of MEF2D protein after *Plaur* induction and confirmed Plaur-miR1-5p-mediated activation of *Mef2d* gene expression in Neuro2a cells. In conclusion, we identified novel small RNAs - Plaur-miR1-3p and Plaur-miR1-5p—encoded in the mouse *Plaur* gene. The obtained results enable an increasingly deeper and more nuanced understanding of *Plaur* gene function in brain development and functioning.

## Materials and Methods

### Bioinformatic Prediction of miRNA in *Plaur* and Its Candidate Target Genes

To identify miRNA in the *Plaur Mus musculus* gene, we employed the following bioinformatic tools. *Promoter 2.0*^2^ and ElemeNT^3^ computational tools were used to detect core promoter elements upon screening RNA polymerase III (Pol III) promoter regions (Sloutskin et al., 2015). Putative miRNA precursors were identified by using the *miRNA Fold* web service^4^ (Supplementary Figure 1; Tav et al., 2016). We selected one from the predicted stem-loop structures based on the stem-loop length, the free energy of the stem-loop formation and consensus motifs enhancing Drosha processing: a basal UG motif, a flanking CNNC motif, a mismatched GHG motif and an apical UGU/GUG motif (Lee and Shin, 2018). Stem-loop structures were visualized by implementing *Quickfold* (Supplementary Figure 2).^5^ In the selected stem-loops, Drosha cleavage sites were predicted by using *MatureBayes* (Gkirtzou et al., 2010) and putative mature miRNAs were identified (Supplementary Figure 3).

To identify the putative targets and binding sites of predicted miRNA, we employed a web-based target prediction algorithm, namely *MR-microT* in *DianaTools* (Supplementary Figure 4A; Reczko et al., 2012; Paraskevopoulou et al., 2013).^6^ The sequences of predicted miRNAs were used as an input. The mouse genome (*Mus musculus*, Ensembl v84) was used as a database for target mRNA prediction (Supplementary Figure 4B). We selected several predicted target mRNAs (Supplementary Tables 1, 2) for further verification. To verify the identified targets, small RNA sequencing using the Plaur-miR1 primer was performed. The obtained sequences were mapped in the *Mus musculus* genome using the BLAST algorithm. Genes that appeared both in the BLAST search and MR-microT prediction were selected for quantitative real-time polymerase chain reaction (qPCR) verification. To assess the specificity of Plaur-miR1-5p and Plaur-mir1-3p to the target gene promoter regions and introns, we took NCBI gene sequences and aligned them against Plaur-miR1-5p and Plaur-mir1-3p via the M-Coffee sequence alignment tool on the T-COFFEE Multiple Sequence Alignment Server web service (Supplementary Figure 4C).

### Cell Culture

Mouse Neuro2a neuroblastoma cells (ATCC^®^ CCL-131™ University Boulevard Manassas, VA, United States) not exceeding 20 passages were cultured in complete medium—Dulbecco’s Modified Eagle’s Medium (DMEM) (#21969035), 10% fetal bovine serum (FBS, Gibco, #10270-106, United Kingdom), 1 × Minimum Essential Medium (MEM) Non-Essential Amino Acids Solution (#11140050) and 1 × antibiotic-antimycotic solution (#15240062; all from Gibco, Life Technologies, Bleiswijk, Netherlands)—at 37^°^C in an atmosphere with 5% CO_2_. Cells were plated at a concentration of 1 × 10^5^ cells/ml. Neuro2a cells with uPAR knockout were obtained by using the CRISPR/Cas9 (Neuro2a KO-*Plaur* cells) genome editing tool as described previously (Rysenkova et al., 2018).

### Animal and Tissue Samples

We had previously shown that *Plaur* gene expression is induced in various brain structures (Shmakova et al., 2020) in a model of pentylenetetrazole (PTZ)-induced seizures in mice. Here we used miRNA and mRNA isolated from previously obtained brain samples. A detailed description of methodology, animal facility and enabling documentation has been previously published (Shmakova et al., 2020). To assess the endogenous level of Plaur-miR1 induction and expression of its target genes, we selected brain regions with the most significant *Plaur* mRNA induction 3 h after PTZ (Sigma-Aldrich, cat. # P6500, Saint Louis, MO, United States) administration. Thus, posterior cortex (*Plaur* induction was 8.7 times higher than control) was enrolled to assess the level of Plaur-mir1-5p and Plaur-mir1-3p and the striatum (*Plaur* induction was 16 times higher than control)—for the target gene analysis (for further information, see Figure 2 in Shmakova et al., 2020).

### Construction of the pBl-U6-Plaur-Pre-miR1 Vector for Overexpression of Plaur-Pre-miR1 in Neuro2a Cells

To clone Plaur-pre-miR1 (precursor of mature Plaur-miR1), we used a vector for mouse Plaur-pre-miR1 expression originally based on the pBl-U6-CMV-RFP (pBlueScriptII vector from Agilent Santa Clara, CA, United States) plasmid and encoding red fluorescent protein (RFP) for detection (Supplementary Figure 5). Mouse Plaur-pre-miR1 was amplified from Neuro2a genomic DNA using primers listed in Supplementary Table 1. For amplification, we used Phusion High-Fidelity PCR Master Mix (#F531L, Thermo Fisher Scientific, Vilnius, Lithuania) according to the manufacturer’s protocol. The program for template denaturation, primer annealing and primer extension was 40 cycles of 94°C for 15 s, 70^°^C for 15 s and 72^°^C for 25 s, respectively. The product length was 280 base pairs (bp). The sequences were cloned into the pBl-U6-CMV-RFP plasmid via *Bbs*I (#R0539, New England Biolabs, Ipswich, MA, United States) restriction sites. The pBl-U6-Plaur-pre-miR1 plasmid sequence was verified using the seq u6 primer 5′-CCTATTTCCCATGATTCCTTCATATTTGC-3′ (Supplementary Figure 6; sequencing was performed by Evrogen, Moscow, Russia).

The pBl-U6-Plaur-pre-miR1 vector was transfected in Neuro2a cells with Lipofectamine 2000 according to the manufacturer’s protocol. The Neuro2a cell transfection efficiency was evaluated basing on the RFP fluorescence analysis 24 h after transfection using Leica DMI 6000 B fluorescent microscope and LAS X software (Wetzlar, Germany). Over a period of 48 h, Neuro2a-Plaur-pre-miR1-transfected cells were lysed and a fraction of small RNAs (mirVana miRNA Isolation Kit AM1560, Ambicon, Carlsbad, CA, United States) and total RNA (Quick-RNA MicroPrep R1051, Invitrogen, Freiburg, Germany) was purified to assess the Plaur-miR1 and its target genes expression level.

### Quantitative Real-Time Polymerase Chain Reaction of Plaur-miR1-5p and Plaur-miR1-3p

Short RNAs (< 200 nt small RNAs including pri-miRNAs, pre-miRNAs and mature miRNAs) were isolated from wild type Neuro2a cells, *Plaur*-deficient cells (Neuro2a KO-*Plaur*) (Rysenkova et al., 2018), Neuro2a cells overexpressing Plaur-pre-miR1 (three replicates per cell group), and from posterior cortex samples (three animals per group) 0 and 3 h after PTZ treatment according to the manufacturer’s protocol (mirVana miRNA Isolation Kit AM1560, Ambicon, Carlsbad, CA, United States). To generate complementary DNA (cDNA), 500 ng of small RNAs fraction and miScript II RT kit (#218160, Qiagen, Hilden, Germany) was used. PCR was carried out by using qPCR mix-HS SYBR (Evrogen) on a CFX96 real-time PCR device (Bio-Rad, Hercules, CA, United States). qPCR was employed to detect Plaur-miR1-5p and Plaur-miR1-3p from Plaur-pre-miR1. Primers for Plaur-miR1 were designed with NCBI Primer-blast^7^ and the IDT Oligo Analyzer tool (eu.idtdna.com/pages/tools/oligoanalyzer). For reverse primers, we used the commercially available 10 × miScript universal primer from the miScript SYBR^®^ Green PCR Kit (#218073, Qiagen, Hilden, Germany). All primers are listed in Supplementary Table 1. The thermal cycling program for template denaturation, primer annealing and primer extension was 40 cycles of 94^°^C for 15 s, 57^°^C for 15 s and 72^°^C for 20 s, respectively. The relative transcript level of Plaur-miR1-5p was calculated using the 2^–ΔΔCt^ method with *Snord95* as a reference; normalization was carried out by taking the average level of each transcript in the control as a unit. The PCR products of Plaur-miR1-5p obtained from Neuro2a cells and posterior cortex were cloned into 40 TA plasmids and subjected to Sanger sequencing (performed by Evrogen).

### Quantitative Real-Time Polymerase Chain Reaction of Target Genes mRNA for Plaur-miR1-5p and Plaur-miR1-3p

Total RNA was extracted from wild type Neuro2a cells, *Plaur*-deficient cells (Neuro2a-KO uPAR) and Neuro2a cells overexpressing Plaur-pre-miR1 (three replicates per each group) (Supplementary Figure 7A) as well as from the striatum of control mice (treated with saline) and mice 0.5, 1, 3, 6, 24, and 72 h after PTZ treatment (4–5 animals per group). The Quick-RNA MicroPrep kit with TRIzol (#R1051, Invitrogen, Carlsbad, CA, United States) was used according to the manufacturer’s protocol. The isolated RNA was treated with RNase-free DNAase I (Fermentas, Rockford, IL, United States) and then run on an agarose gel for quality control (Supplementary Figure 7E). To generate cDNA, 1μg of total RNA and the MMLV RT kit (Evrogen) were used. PCR was carried out using qPCR mix-HS SYBR (Evrogen) and the CFX96 Touch Real-Time PCR Detection System (Bio-Rad). qPCR was used to detect the expression of Plaur-miR1-5p target genes. Primers were designed using NCBI Primer-blast (see text footnote 8) and the IDT Oligo Analyzer tool.^8^ All primers are listed in Supplementary Table 1; the *Plaur* mRNA primers are specific to exon 4. The thermal cycling program for template denaturation, primer annealing and primer extension was 40 cycles of 94°C for 10 s, 60°C for 30 s, and 72°C for 15 s, respectively. The relative transcript level of mRNA was calculated using the 2^–ΔΔCt^ method with *Actb* (encodes β-actin) as a reference. The reactions were performed in instrumental triplicates; the results represent the mean of biological triplicates (unless otherwise stated) ± standard error of the mean (SEM).

### Western Blot

Brain tissue samples was homogenized and lysed in an ice-cold RIPA lysis buffer as previously described (Shmakova et al., 2020). Proteins (45 μg) were resolved in 10% SDS-PAGE gels and transferred to PVDF membrane (GE Healthcare) in the transfer buffer (25 mM Tris, 192 mM glycine, 0.1% SDS and 20% methanol). Non-specific binding was blocked by 5% non-fat dried milk in phosphate buffered saline (PBS, Sigma-Aldrich), containing 0.1% Tween-20 at + 4°C overnight. Proteins were probed with the following primary antibodies in 1:1,000 dilution: rabbit anti-SNRNP200 (Sigma, HPA029321), rabbit anti-MEF2D (Cell signaling, 25621), rabbit anti-EMX2 (Abcam, ab94713), rabbit anti-β-actin (Cell signaling, 4970S, control of protein load) for 2h at room temperature. Membranes were washed with PBS containing 0.1% Tween-20 and incubated with appropriate peroxidase-conjugated secondary antibodies in 1:10,000 dilution for 1.5h at room temperature, followed by washing in PBS containing 0.1% Tween-20. Proteins were visualized using SuperSignal West Dura Chemiluminescent Substrate (Thermo Fisher Scientific) and ChemiDoc™ XRS + System (Bio-Rad) for Western blotting imaging and analysis. Densitometric analysis of blots at non-saturating exposures was performed using ImageJ. Values of SNRNP200, MEF2D, and EMX2 protein expression were normalized to β-actin. Original uncropped western blot images are presented in Supplementary Figure 10.

### Luciferase Reporter Assay

DNA fragments encoding the predicted binding sites for Plaur-miR1-5p (3′UTR for Mef2d [positions 3376-3499 and 5140-5276 in NM_001310587.1], *Emx2* [positions 2191-2281 in NM_010132.2] and CDS for *Snrnp200* [positions 3037-3114 in NM_177214.5]) were amplified from murine cDNA (see Supplementary Table 1 for primers) using Phusion High-Fidelity PCR Master Mix (Thermo Fisher Scientific, # F531L) and inserted in pGL3-promoter vector (Promega, #U47298). *Hin*dIII and *Nco*I restriction sites were used for CDS sequences, while *Xba*I and RigI restriction sites were used for 3′-UTR. The sequence of the resulting vectors was confirmed using Sanger sequencing.

Neuro2a cells were seeded onto 96-well plate, cultured into a monolayer, and co-transfected with PGL3 plasmids encoding *Mef2d* 3′-UTR sequences, *Emx2* 3′-UTR sequence, *Snrnp200* CDS sequence or empty pGL3 vector (control) with plasmid pBl-U6-Plaur-pre-miR1 or pBl-U6 vector. 48 h after transfection cells were analyzed using Luciferase Reporter Assay Kit (Promega, Fitchburg, WI, United States). Luminescence was evaluated using Victor™ X3 Multilabel Plate Reader (Perkin-Elmer Inc., United States), the luciferase signal was calculated in Relative luciferase activity units. Data were normalized by luciferase signal in Neuro2a cells co-transfected with empty pGL3 vector and pBl-U6-Plaur-pre-miR1 vector.

### Statistical Analysis

We analyzed qPCR data with GraphPad Prism 8.01 (GraphPad Software Inc., San Diego, CA). For cells we analyzed qPCR data using an unpaired *t*-test, treating the wild type and experimental cells as two independent groups. For comparisons with more than two groups, we used analysis of variance (ANOVA) followed by Dunnett’s multiple comparisons test. Relative luciferase activity data were compared using two-way ANOVA followed by Šídák’s multiple comparisons test. The data are presented as the mean ± SEM. The level of significance was set at *P* < 0.05.

## Results

### Computational Prediction of a Novel miRNA in the *Mus musculus Plaur* Gene and Its Target Genes

We first hypothesized that small RNAs are encoded within *Plaur* as individual genes under specific Pol III promoters. Bioinformatic screening of *Plaur* employing ElemeNT (Sloutskin et al., 2015) and Promoter 2.0 failed to reveal any specific binding sites for Pol III. Hence, no predicted transcription start sites (TSS) for miRNA genes within *Plaur* that could give rise to miRNAs via the classical biogenesis pathway were identified. Since there is considerable evidence indicating that miRNAs can be located in the introns of protein-coding genes—called mirtrons (Dokanehiifard et al., 2015, 2017)—we next tested the hypothesis that *Plaur* can contain such structures. The sequences of predicted miRNAs are summarized in Table 1.

**TABLE 1:** Selected precursor miRNAs encoded in the *Mus musculus Plaur* gene.

Name	Sequence (5′→3′)	Length	Genome position
Plaur-pre-miR1	GGACUUGGGAUAAGUAGGCUUGGUGAUUGGCUGCCAGGUUCAGAGUGGAGUUCUCUGCAGGACCU GGCCGCCAACACGCCACUCUCUCCUCUUCCUAGGAGCC	103	24,171,226–2,417,132
Plaur-pre-miR2	GUAGGUGGAUCUCUGGGUUUGAGGACAGCCUGGUCCAUACAGAGAGGCCCUGUCUGGGGGGUGGG GAGGAGGCGGUGUCUACCUGC	86	24,173,938–24,174,023
Plaur-pre-miR3	AAGAGGGGUGGGACAGACAGCGUGGCUGUGCUGGAAAUUCCUGUUUUGAUUUUUUUUCCCCCAAG ACAGGGUUUCUCUGUAUAGCCCCUGGCUGUCCUGGAACUCACUU	109	24,169,492–24,169,600

**FIGURE 1:**
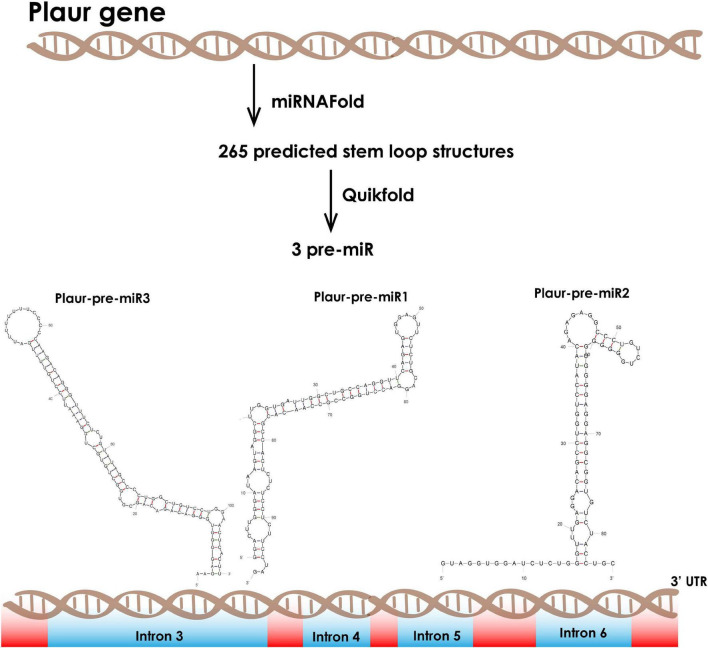
Computational prediction of novel small RNAs in the *Mus musculus Plaur* gene. We applied the miRNA Fold software to predict miRNA precursor hairpin structures located in *Plaur*. We then used the Quickfold service for subsequent visualization and analysis of 256 predicted stem-loops. We identified three stem-loop structures with high probability to be miRNAs according to the intronic localization, overall stability (≤ −15 kJ/mol) and consensus motifs that are critical for Drosha processing. Plaur-pre-miR1 and Plaur-pre-miR3 are located in intron 3 and Plaur-pre-miR2 is located in intron 6. Introns are highlighted in blue; exons are highlighted in red.

We employed the miRNA Fold^9^ web-based prediction service to reveal miRNA precursors in *Plaur* (GRCm39, Gene ID: 18793). miRNA Fold predicted 256 stem-loop structures, which are potential miRNA precursors, located within *Plaur*. We filtered the most stable predicted stem-loops with free energy of formation ≤ −15 kJ/mol (Xue et al., 2005). Such structures occur infrequently, although they are frequently non-random because evolution should theoretically reject them. Among that putative pre-miRNA, three ones had a high probability to exist *in vivo* based on motifs enhancing Drosha processing: Plaur-pre-miR1 (located in intron 3), Plaur-pre-miR2 (located in intron 3) and Plaur-pre-miR3 (located in intron 6) (Figure 1 and Table 1).

**FIGURE 2:**
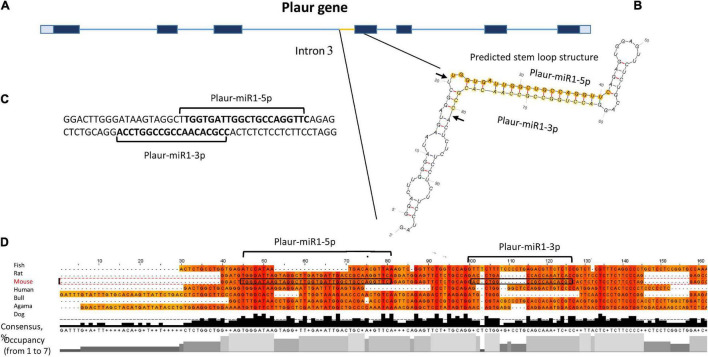
Identification of novel small RNAs Plaur-miR1: Plaur-miR1-3p and Plaur-miR1-5p in the *Mus musculus Plaur* gene. **(A)** Schematic overview of mouse *Plaur* gene, adapted from NCBI Genome browser. Untranslated regions are shown in gray. Exons and introns are indicated as blue rectangles and lines, respectively. **(B)** The predicted stem-loop structure of Plaur-miR1 from *Plaur* intron 3. Arrows indicate predicted sites of Drosha cleavage; mature miRNAs from the stem-loop, namely Plaur-miR1-5p and Plaur-miR1-3p, are highlighted in orange and yellow, respectively. **(C)** The duplex part of the stem-loop is indicated; sequences of mature Plaur-miR1-5p and Plaur-miR1-3p predicted by *MatureBayes* are shown in bold (predicted Drosha cutting). **(D)** The intronic region corresponding to the novel miRNA in the *Plaur* gene is highly conserved among vertebrates. Multiple sequence alignment with Clustal Omega (visualization Jalview 2.11.0) revealed consensus regions matched to mature Plaur-miR1-5p. Areas corresponding to the sequences of mature miRNAs are circled in frames; vertebrate species are indicated on the right. The consensus diagram shows the occurrence of one nucleotide in a given position; the occupancy diagram shows the number of nucleotides in a given position.

We focussed on Plaur-pre-miR1 since it contains three motifs that enhance Drosha processing (Lee and Shin, 2018), namely the 5′ GUG motif on the apical loop, an unpaired GHG in the downstream part of the stem and a UG motif in the base of the stem-loop structure. It is located upstream of exon 4 (Figure 2A), with free energy of formation −47.6 kJ/mol, indicating its high stability. Due to the presence of these consensus sequences critical for Drosha processing activity, we anticipated a high probability of Plaur-pre-miR1 being a substrate for Drosha processing (Figure 2B).

Hence, we conducted *in silico* Drosha cleavage of Plaur-pre-miR1 to locate the miRNA precursor within its sequence. We predicted Drosha cleavage sites as well as mature miRNA sequences using *MatureBayes*. According to the processing rules of the Drosha enzyme, the mature miRNA sequences located at the hairpin 5′ end (5p miRNAs) and predicted *in silico* corresponded to those predicted by the *MatureBayes* program (Supplementary Figure 3). However, the sequences located at the 3′ end (3p miRNAs) were mis-predicted by the *MatureBayes* program since they were located at the apical loop region therefore contradicting the Drosha-mediated processing mechanisms. Hence, we predicted 3p miRNA sequences following the Drosha processing rules: to protrude two nucleotides at the 3′ end of each mature miRNA (Figure 2C).

To verify Plaur-miR1-5p and Plaur-miR1-3p sequences predicted by *MatureBayes* and to confirm our predictions based on the Drosha processing pattern, we performed multiple sequence alignment of the *Plaur* region corresponding to Plaur-pre-miR1 in different vertebrate species: *Bos taurus*, *Mus musculus*, *Canis lupus familiaris*, *Homo sapiens*, *Pogona vitticeps*, and *Poecilia latipinna* (Figure 2D). Similar alignment results for Plaur-miR2 and Plaur-miR3 are presented in Supplementary Figure 8. The region corresponding to Plaur-miR1-5p showed maximum occupancy in possible miRNA seed sequence regions; together with a high consensus percentage (50–90%) the Plaur-miR1-5p sequence appeared to be conserved among all analyzed vertebrate species. There was also a TG sequence complementary to UG in RNA among all species in a highly homologous region with respect to the hairpin base. This is consistent with the literature on consensus sequences contributing to the Drosha/DGCR8 microprocessor complex function, located approximately 13 nt downstream the Drosha 5p cleavage site (Auyeung et al., 2013). In contrast, low occupancy and consensus ≤ 50% indicate that the Plaur-miR1-3p sequence was not conserved. Thereby, we assumed that Plaur-miR1-3p is a passenger strand and Plaur-miR1-5p is a novel guide strand. Although Plaur-miR1-5p showed similarity to mmu-miR-7672-3p in miRDataBase encoded within the gene *PDE12* (Chr14:26390702-26390763 bp, GRCm39,—strand, according to Blast alignment), an identical miRNA for Plaur-miR1 has not yet been reported in miRbase. Overall, the high Plaur-miR1-5p sequence homology among the analyzed organisms suggests that it has been a subject to natural selection due a potentially important biological function. Assumingly, the 5p mature form performs a guiding function, while the 3p form degrades. In this regard, we have focused primarily on Plaur-miR1-5p and its targets.

Next, using the DianaTools (v84) web server, we performed computational prediction of Plaur-miR1-5p and Plaur-miR1-3p target genes. The following mature miRNA sequences were entered into the search bar: Plaur-miR1-5p 5′-UGGUGAUUGGCUGCCAGGUUC-3′ and Plaur-miR1-3p 5′-AGAACCUGGCCGCCAACA-3′. The search results are presented in Supplementary Table 2 for Plaur-miR1-5p and Supplementary Table 3 for Plaur-miR1-3p, as well as in primary screenshots of the web portal (Supplementary Figure 4B). Since miRNAs engage with various targets, 800 Plaur-miR1 targets were obtained, among which 50 targets had a binding score > 0.9 (set as the screening threshold).

**FIGURE 3:**
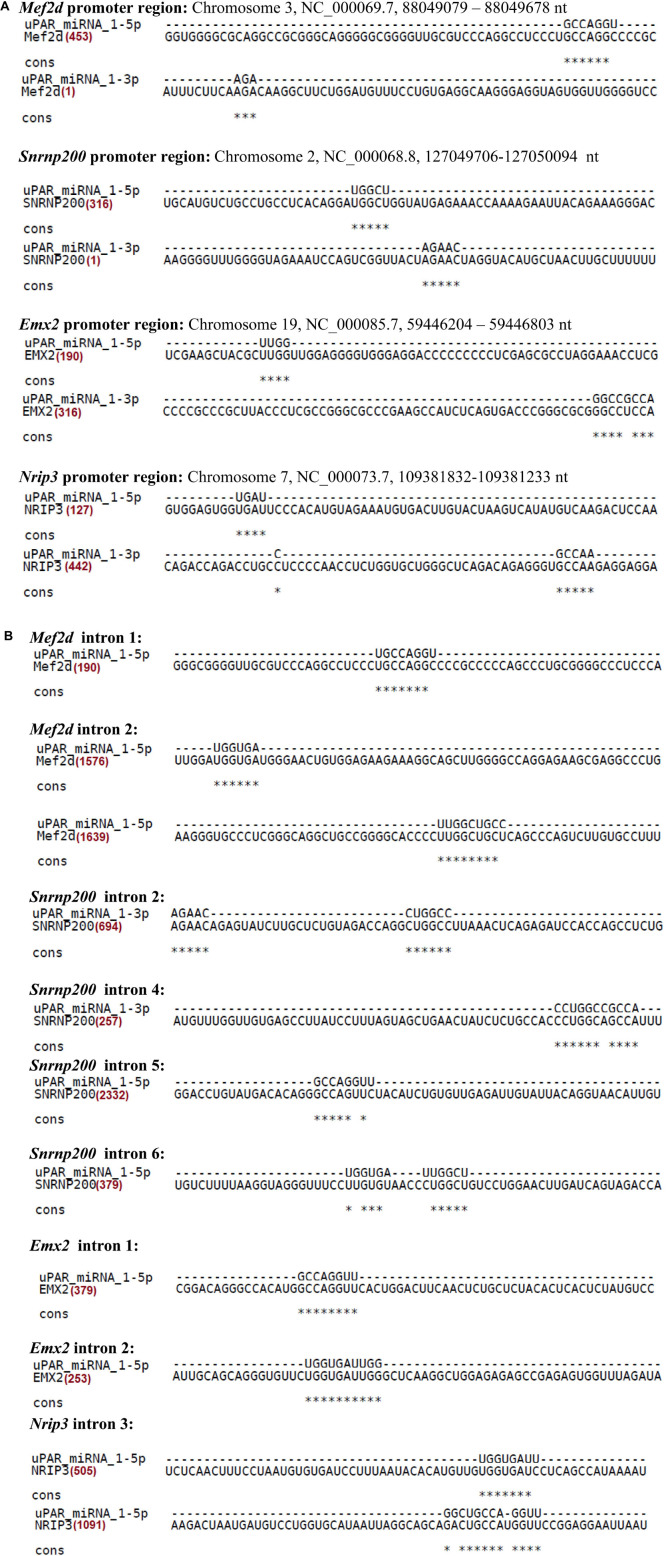
Alignment of Plaur-miR1-5p **(A)** and Plaur-miR1-3p **(B)** with promoter regions and introns of the predicted target genes *Mef2d*, *Emx2*, *Snrnp200*, and *Nrip3* with the M-coffee web service T-COFFEE Multiple Sequence Alignment Server.

We have previously shown that uPAR plays a role in neuronal cell differentiation and survival (Rysenkova et al., 2020). Moreover, we have found a correlation between high expression of uPAR and induction of neuronal migration to the outer layers of cerebral cortex, as well as *Plaur* function as an early response gene in the brain, a characteristic that possibly determines uPAR as a morphogen (Shmakova et al., 2021). Hence, we analyzed the possible targets obtained by DianaTools to study in further detail the role of Plaur-miR1-5p and Plaur-miR1-3p with a special focus on targets potentially involved in neuronal differentiation or surveillance, as well as the maturation of brain structures. *Emx2* (empty helix homeobox 2) was the target for Plaur-miR1-5p with the highest score (0.998). *Emx2* is a transcription factor that plays a pivotal role in the developing brain, determining cell fate in the embryonic central nervous system (Gulisano et al., 1996). For Plaur-miR1-3p, *Mef2d* (myocyte enhancement factor 2D) was a target with a highest score (0.989). Mef2d is a transcription activator that plays a key role in the regulation of neuronal apoptosis (Wang et al., 2009; Assali et al., 2019). Moreover, *Mef2d* also was a target for Plaur-miR1-5p with a score (0.485) as detected by DianaTools (Supplementary Figure 4C). Therefore, we analyzed *Emx2* and *Mef2d* mRNA expression in Neuro2a control cells, Neuro2a KO-*Plaur* and Neuro2a-Plaur-miR1 cells (ectopic Plaur-miR1 expression) to assess the impact of Plaur-miR1-5p and Plaur-miR1-3p on their targets. Moreover, Plaur-miR1-5p and Plaur-miR1-3p have a recognition site in the *Emx2* and *Mef2d* gene promoters (Figure 3A) and introns (Figure 3B and Supplementary Figure 4C), suggesting that Plaur-miR1 regulates the expression of these target genes at the nuclear level, including their expression induction. To examine the Plaur-miR1-5p specificity to the promoter region [600 nt upstream of the TSS, according to Ensembl genome browser]^10^ and target gene introns, we used intronic sequences from NCBI and aligned them with Plaur-miR1-5p and Plaur-miR1-3p via the M-Coffee tool of the T-COFFEE Multiple Sequence Alignment Server web service.

### Detection of Plaur-miR1-5p and Plaur-miR1-3p in Mouse Neuro2a Cells and Mouse Brain

The obtained bioinformatic data on the existence of murine Plaur-miR1-5p and Plaur-miR1-3p was verified in Neuro2a cells and posterior cortex of C57BL/6J mice. Since the studied miRNAs are located in *Plaur* introns we used previously obtained Neuro2a cells with CRISPR/Cas9n-mediated *Plaur* knockout (Neuro2a KO-*Plaur*) (Rysenkova et al., 2018; Semina et al., 2020) as a negative control and wild type Neuro2a cells (Neuro2a WT) endogenously expressing *Plaur* for measuring the miRNA levels in Neuro2a cells.

**FIGURE 4:**
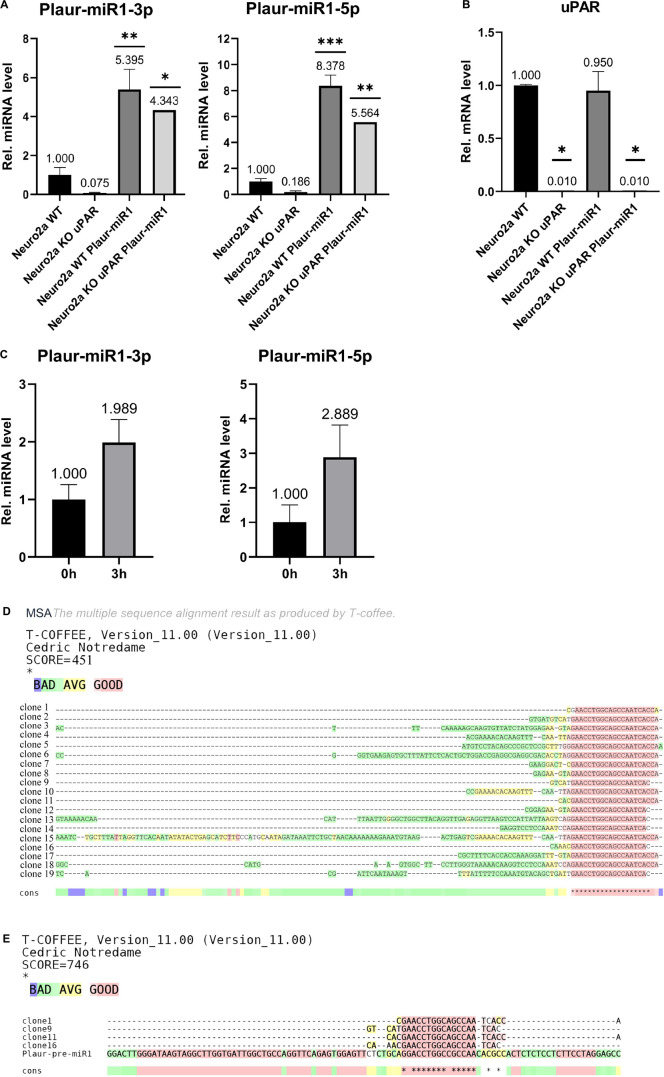
Analysis of Plaur-miR1-3p and Plaur-miR1-5p expression in Neuro2a cells and mouse posterior cortex. **(A)** qPCR of Plaur-miR1-3p and Plaur-miR1-5p expression in Neuro2a cells. The data are expressed as mean ± SEM (*n* = 3), normalized to *Snord95* expression as a reference gene. **(B)** qPCR of *Plaur* expression in Neuro2a cells. The data are expressed as mean ± SEM (*n* = 3), normalized to *Actb* (encodes β-actin) expression as a reference gene. For **(A,B)**, the columns are: Neuro2a WT—control Neuro2a cells; Neuro2a-KO-uPAR—uPAR-deficient Neuro2a cells; Neuro2a Plaur-miR1—Neuro2a cells with ectopic Plaur-pre-miR1 expression; Neuro2a-KO-uPAR Plaur-miR1—uPAR-deficient Neuro2a cells with ectopic Plaur-pre-miR1 expression. The data were analyzed by using analysis of variance followed by Dunnett’s multiple comparisons test using GraphPad Prism software. **(C)** qPCR of Plaur-miR1-5p and Plaur-miR1-3p in the posterior cortex 0 and 3 h after endogenous *Plaur* induction. The data are expressed as mean ± SEM (*n* = 4). The data were analyzed by using a one-sample *t*-test with GraphPad Prism software. Statistical significance in **(A–C)** is indicated by bars and asterisks as follows: *p < 0.05; ^**^p < 0.01; ^***^p < 0.001. **(D)** Sequence results of 19 TA vector clones containing Plaur-miR1-5p sequences generated in **(C)**. Four of 19 (20%) clones were 22–24 bp in length and could correspond to mature miRNA. In addition, 8 of 19 (40%) clones were 22–31 bp in length and could correspond to other RNA fragments isolated from posterior cortex small RNA fraction (Supplementary Figure 7D). **(E)** Clones 1, 9, 11, and 16 show the Plaur-miR1-5p sequence, demonstrated as alignment with Plaur-pre-miR1. The asterisks in **(D,E)** indicate that the aligned sequences match at that position.

Plaur-miR1-5p and Plaur-miR1-3p were detected in Neuro2a WT cells but not in Neuro2a KO-*Plaur* cells (Figure 4A). These findings suggest that these miRNAs are expressed in Neuro2a cells and their expression level is *Plaur* dependent. Moreover, transfection of Neuro2a cells with the pB1-miR plasmid to overexpress Plaur-miR1-5p and Plaur-miR1-3p increased the content of these miRNAs in Neuro2a cells (Neuro2a WT Plaur-miR1 cell in Figure 4A) and restored their expression in Neuro2a KO-*Plaur* cells (Neuro2a KO WT Plaur-miR1 cell in Figure 4A). *Plaur* mRNA expression was verified by qPCR (Figure 4B). The specificity of the observed effects (Figure 4A) was further confirmed by the lack of change in uPAR expression level in Neuro2a cells transfected with the pB1-miR plasmid for Plaur-miR1-5p and Plaur-miR1-3p overexpression (Neuro2a WT Plaur-miR1 and Neuro2a KO uPAR Plaur-miR1 cells in Figure 4B).

Subsequently, we analyzed the Plaur-miR1-5p and Plaur-miR1-3p expression in the mouse brain (posterior cortex) in control conditions and 3 h after PTZ-induced *Plaur* expression (Shmakova et al., 2020). Surprisingly, we revealed not only Plaur-miR1-5p and Plaur-miR1-3p expression in the posterior cortex, but their expression was increased by 1.9 and 2.6 times, respectively, after PTZ treatment (Figure 4C).

qPCR using a small RNA matrix (< 200 nt) with primers for Plaur-miR1-5p yielded a single product with an approximate size of 50 nt (Supplementary Figure 9). To confirm the specificity of qPCR performed with Plaur-miR1-5p primers and to establish the nucleotide sequence of all PCR products in this reaction, we sequenced the qPCR product with the Plaur-miR1-5p primers using miRNA samples from posterior cerebral cortex. Sequencing of PCR products and their cloning into TA vector was carried out by Evrogen. The results of 19 vector clones of TA containing Plaur-miR1-5p sequences are shown in Figure 4D. Clones 1, 9, 11, and 16 demonstrate the sequence similarity in 15 out of 22 nucleotides to Plaur-miR1 (Figure 4E) and are 22–24 nt in length. All other sequences range in size from 31 to 134 nt suggesting that they may be related to other RNA fragments in small RNA fraction (< 200 nt).

The sequencing results revealed that only 20% (4 out of 19 clones) were the target products of Plaur-miR1-5p. Actually, an accurate detection of the relative Plaur-miR1-5p expression in posterior cortex may not be feasible at this stage and may be masked due to the presence of by-products. One of the possible reasons would be different levels of the *Plaur* and Plaur-miR1-5p induction (8.8-folds for *Plaur*, refer to Figure 2 in Shmakova et al. (2020); 2.9-folds for Plaur-miR1-5p (Figure 4C) in posterior cortex. Beyond that, the induction difference could stem from rapid degradation of Plaur-miR1-5p in the cytoplasm due to interaction with its targets (Agrawal et al., 2003).

Nevertheless, the qPCR product sequencing results allowed us to determine the possible targets for Plaur-miR1-5p using experimental approach. For that, we aligned the obtained sequencing products > 31 nt in length against *Mus musculus* genome GRCm39 using the BLAST algorithm. Two genes appeared in the BLAST search: *Snrnp200* (U5 small nuclear ribonucleoprotein) and *Nrip3* (nuclear receptor interacting protein 3). We then analyzed the mRNA expression levels of these genes by qPCR. Of note, both *Snrnp200* and *Nrip3* had rather low score values according to the prediction of targets in DianaTools: 0.48 and 0.38, respectively.

### Evaluation of the Level of Predicted Target Genes of Plaur-miR1-3p and Plaur-miR1-5p

**FIGURE 5:**
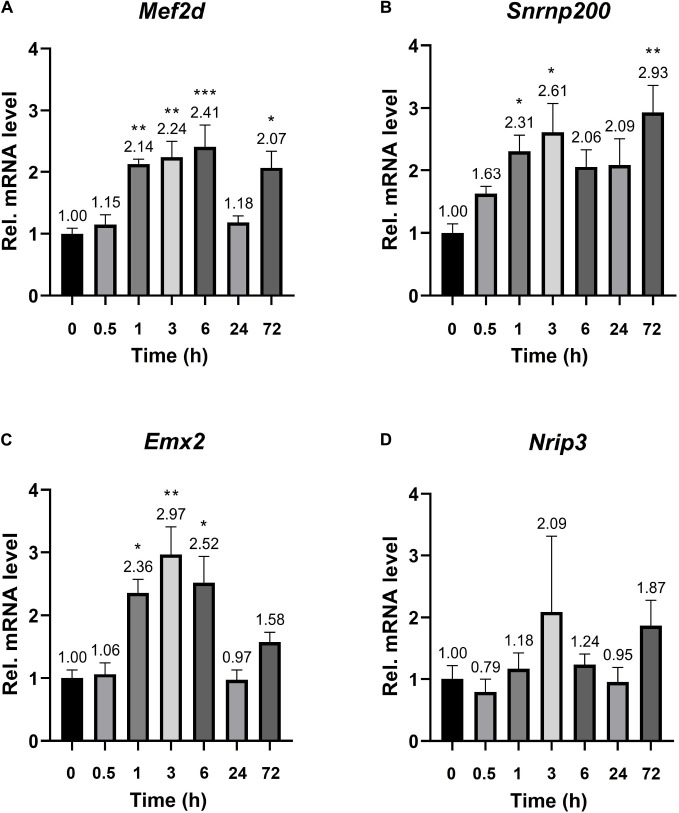
mRNA expression of the Plaur-miR1-3p and Plaur-miR1-5p target genes *Mef2d*, *Snrnp200*, *Emx2*, and *Nrip3* in mouse striatum. qPCR analysis of **(A)** *Mef2d*, **(B)** *Snrnp200*, **(C)** *Emx2* and **(D)** *Nrip3* expression in the striatum of control mice (treated with saline, 0 h) and mice 0.5, 1, 3, 6, 24, and 72 h after endogenous *Plaur* induction. The data are presented as mean ± SEM (*n* = 4), normalized to *Actb* (encodes β-actin) expression as a reference gene. The data were analyzed by using analysis of variance followed by Dunnett’s multiple comparisons test with GraphPad Prism software. Statistical significance is indicated by bars and asterisks as follows: *p < 0.05; **p < 0.01; ***p < 0.001.

The established *Plaur*-dependent expression of Plaur-miR1-3p and Plaur-miR1-5p in the mouse brain prompted us to evaluate the expression of Plaur-miR1-3p and Plaur-miR1-5p target genes—*Emx2* and *Mef2d* identified via DianaTools—as well as *Snrnp200* and *Nrip3*, identified in qPCR products from posterior cortex samples. The target gene expression was assessed in the striatum, which, as we have previously reported, exhibited the highest increase in *Plaur* expression after PTZ treatment: 16 times compared with the control group, as presented in Figure 3 in the paper by Shmakova et al. (2020). Mouse striatum samples were enrolled for analysis at the baseline and 0.5, 1, 3, 6, 24, and 72 h after PTZ administration. We found a significantly increased expression of *Mef2d*, *Emx2*, and *Snrnp200* mRNA (Figures 5A–C). Moreover, the induction dynamics was consistent with the *Plaur* expression dynamics after PTZ treatment, as shown in Figure 2 by Shmakova and co-authors (Shmakova et al., 2020). Meanwhile, the *Mef2d* and *Snrnp200* expression remained elevated by more than 2-folds 72 h after PTZ treatment. *Emx2* expression was elevated up to for 6 h after PTZ treatment, with a maximum increase of 2.97-folds after 3 h as compared with endogenous *Plaur* expression. *Nrip3* expression remained unchanged at all the tested time points (Figure 5D).

**FIGURE 6:**
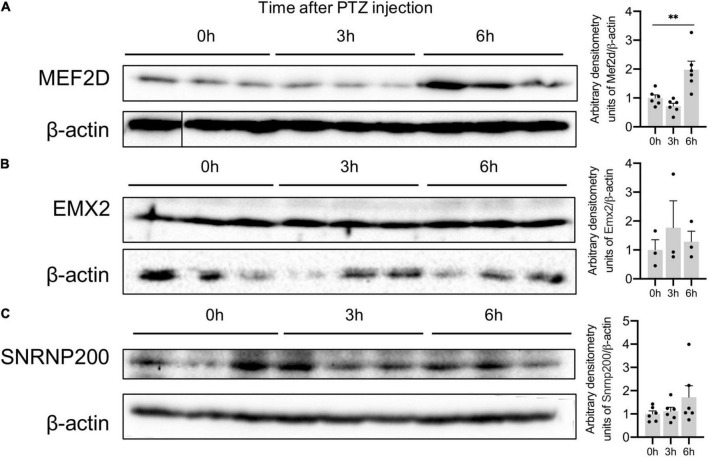
Protein expression of Plaur-miR1 (Plaur-miR1-3p and Plaur-miR1-5p) target genes *Mef2d*, *Snrnp200*, and *Emx2* in mouse striatum. Western blot analysis of **(A)** MEF2D, **(B)** SNRNP200 and **(C)** EMX2 expression in striatum of control mice (treated with saline, 0 h) and of mice after 3 and 6 h induction of endogenous *Plaur* expression. Densitometric analyses and values of protein expression normalized to β-actin are presented as mean ± SEM (*n* = 3–6). The vertical line in **(A)** indicates where the WB lanes were cut and the lanes of the same immunoblot were placed together to allow for a direct comparison. The data were analyzed using analysis of variance followed by Dunnett’s multiple comparisons test with GraphPad Prism software. Statistical significance is indicated by bars and asterisks as follows: ^**^p < 0.01.

We also confirmed an increased expression of MEF2D protein in the mouse brain: MEF2D expression peaked 6h after PTZ administration (Figure 6A). The level of SNRNP200 and EMX2 proteins remained unchanged 3 and 6 h after PTZ administration (Figures 6B,C) suggesting that these genes may be subject to a different transcriptional and translational regulation, which requires further investigation.

**FIGURE 7:**
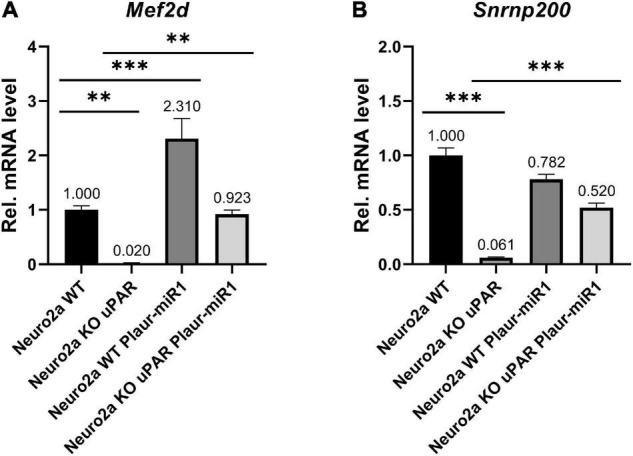
Plaur-miR1 (Plaur-miR1-3p and Plaur-miR1-5p) expression altered the expression of the predicted target genes *Mef2d* and *Snrnp200* in Neuro2a cells. qPCR analysis of **(A)** *Mef2d* and **(B)** *Snrnp200* in Neuro2a WT, Neuro2a KO uPAR, Neuro2a WT, and Neuro2a KO uPAR cells after pBl-U6-Plaur-pre-miR1 expression. The data presented as mean ± SEM (*n* = 3), normalized to *Actb* (encodes β-actin) expression as a reference gene. The data were analyzed by using analysis of variance followed by Dunnett’s multiple comparisons test with GraphPad Prism software. Statistical significance is indicated by bars and asterisks as follows: ^**^p < 0.01; ^***^p < 0.001.

To further expolore the relationship between the expression of Plaur-miR1 and its target genes, we analyzed *Nrip3*, *Snrnp200*, *Emx2*, and *Mef2d* mRNA expression in relation to *Plaur* and Plaur-pre-miR1 expression in Neuro2a cells using qPCR. *Plaur* knockout markedly reduced *Mef2d* and *Snrnp200* expression compared with Neuro2a WT cells: 50-folds for *Mef2d* and 16.4-folds for *Snrnp200* (Neuro2a KO cells in Figure 7). *Emx2* and *Nrip3* expression in the Neuro2a cell was below the detection level. Neuro2a control cells transfected with the pBl-U6-Plaur-pre-miR1 plasmid to overexpress Plaur-miR1-3p and Plaur-miR1-5p showed significantly increased *Mef2d* expression but not *Snrnp200* (Neuro2a WT Plaur-miR1 cells in Figure 7). Moreover, the Plaur-miR1-3p and Plaur-miR1-5p expression restored *Mef2d* and *Snrnp200* expression (in Neuro2a KO uPAR Plaur-miR1 cells in Figure 7), indicating that the expression of the Plaur-miR1-3p and Plaur-miR1-5p targets in Neuro2a cells is strongly dependent on the expression of *Plaur* and Plaur-miR1-3p and Plaur-miR1-5p transcribed from the *Plaur* gene. Thus, we have identified two small RNAs, Plaur-miR1-3p and Plaur-miR1-5p, encoded in intron 3 of the *Plaur* gene, as well as the Plaur-miR1-3p and Plaur-miR1-5p target genes—*Emx2*, *Mef2d*, and *Snrnp200*.

### Direct Interaction of Putative Plaur-miR1-5p With Mef2d, Emx 3′-UTR and CDS of Snrp200

**FIGURE 8:**
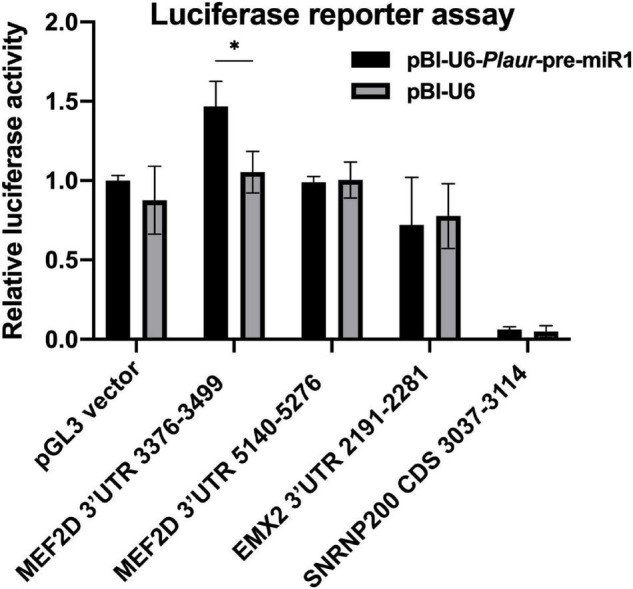
Results of the luciferase reporter assay for the confirmation of specificity of Plaur-miR1-5p effect on its predicted targets. Neuro2a cells were co-transfected with pBl-U6-Plaur-pre-miR1 and pGL3 vector coding 3-UTR or CDS sequences. Empty vectors pBl-U6 and pGL3 were used as controls. Data were normalized by luciferase activity in Neuro2a cells co-transfected with pBl-U6-Plaur-pre-miR1 and pGL3 vectors. The data are presented as mean ± SEM (*n* = 3) and compared using two-way ANOVA followed by Šídák’s multiple comparisons test with GraphPad Prism software. Statistical significance is indicated by bars and asterisks as follows: *p < 0.05.

Since the sequencing of PCR products in cerebral cortex of murine brain (posterior cortex) with primers for Plaur-miR1-5p revealed Plaur-miR1-5p expression, we next addressed if there is a direct interaction between Plaur-miR1-5p and its target genes Mef2d, Snrnp200 and Emx2 . Using DianaTools (v84) web server we predicted microRNA response elements (MRE) for the Mef2d and Emx2 genes in their 3′-UTRs and for the Snrnp200 gene in its CDS sequence (Supplementary Figure 11). We cloned 3′-UTR for Emx2, Mef2d and CDS for Snrnp200 into a pGL3-reporter vector downstream of Renilla luciferase ORF and co-transfected Neuro2A cells with these constructs and pBL-Plaur-pre-miR1 plasmid; pBl-control vector was used as a control (Figure 8). We found that co-transfection of PGL3 vector containing Mef2d 3′-UTR sequence (positions 3376-3499 in NM_001310587.1) with pBL-Plaur-pre-miR1 resulted in a significant ∼1.4-fold increase in luciferase activity (Figure 8, *p* = 0.0178) compared to co-transfection with pBl-control vector. Co-expression of pBL-Plaur-pre-miR1 plasmid with pGL3-reporter vectors containing 3′-UTR sequence of Mef2d outside the mentioned region (positions 5140-5276 in NM_001310587.1), 3′-UTR sequence of Emx2 (positions 2191-2281 in NM_010132.2) or CDS sequence of Snrnp200 (positions 3037-3114 in NM_177214.5) revealed no significant change in luciferase activity compared with pBl-control vector (Figure 8). These results confirmed that Plaur-miR1-5p can specifically increase the Mef2d expression through its 3′-UTR site.

## Discussion

uPAR is a multifunctional, GPI-anchored protein that regulates important processes such as gene expression, cell proliferation, adhesion, migration, invasion and development. Since uPAR lacks membrane and intracellular domains but is anchored to the outer plasma membrane leaflet, it is capable of lateral interactions with other receptors modulating their intracellular signaling pathways. More than 30 signaling proteins have been shown to interact directly with uPAR as lateral partners (Eden et al., 2011). The fact that uPAR interacts with G protein-coupled receptors, receptor tyrosine kinases and integrins implies that the actual number of components constituting the uPAR interactome is exceptionally high. Previous studies revealed cognitive disorders and defects in the development of GABAergic interneurons in uPAR null mice (Powell et al., 2003). uPAR overexpression markedly stimulated the radial neuronal migration to the outer layers of differentiating cortex (Shmakova et al., 2021), which implies uPAR participation in neocortex embryonic development.

Emerging evidence indicates that uPAR is involved in various physiological and pathological conditions in the nervous system, but the known molecular mechanisms of uPAR action do not explain the phenomena. For example, polymorphisms of *Plaur* and the uPAR ligand sushi repeat containing protein X-linked 2 (encoded by the *SRPX2* gene) correlate with human diseases such as epilepsy, autism, multiple sclerosis, Alzheimer’s disease and brain tumors. In addition, we have recently demonstrated that *Plaur* is an early response gene in the mouse brain, which is activated upon PTZ treatment (Shmakova et al., 2020). These data change the conceptual landscape model of uPAR protein and *Plaur* gene functions and expand our knowledge on their role in health and disease. uPAR unexpectedly became a meaningful receptor in the central nervous system, which has opened new perspectives for understanding the embryonic development, normal functioning and pathological changes in the central nervous system. Recent studies have unveiled the myriad roles of miRNAs, highlighting the biological significance of these previously “overlooked” RNA species. In this regard, we hypothesized that previously unknown miRNAs located in the Plaur intronic sequences may determine numerous effects that cannot be otherwise explained by the function of uPAR protein. Indirect evidence supporting this hypothesis resides in the fact that Plaur knockout decreases the *Ntrk3* mRNA expression (encodes TrkC) in mouse Neuro2a cells (Rysenkova et al., 2018).

We conducted a Drosha processing bioinformatic search and found hairpins previously unidentified in the *Plaur* gene structure. We named these pre-miRs Plaur-pre-miR1 (located in intron 3), Plaur-pre-miR2 (located in intron 3) and Plaur-pre-miR3 (located in intron 6) (Figure 1 and Table 1). We focussed on the first hairpin, Plaur-pre-miR1 and proved that the mature miRNAs Plaur-miR1-3p and Plaur-miR1-5p are expressed in Neuro2a cells and mouse brain. Further, we enrolled cells with complete *Plaur* knockout—which we had described previously (Rysenkova et al., 2018)—as well as the brain samples with endogenous *Plaur* induction (Shmakova et al., 2020), obtained as a result of PTZ treatment. The maximal *Plaur* induction was detected in these brain samples by 3 h after PTZ treatment. Here, we demonstrate that Plaur-miR1-3p and Plaur-miR1-5p expression is subjected to *Plaur* expression regulation: Plaur-miR1-3p and Plaur-miR1-5p expression is downregulated upon *Plaur* knockout, while *Plaur* overexpression results in elevated Plaur-miR1-3p and Plaur-miR1-5p expression. Figure 4 demonstrates that Plaur-miR1-5p expression was up to 1.6-folds higher than the expression of Plaur-miR1-3p, assumingly reflecting increased stability of Plaur-miR1-5p in Neuro2a cells (Figures 4A,B).

We used two approaches to identify potential Plaur-miR1 target genes. Based on the collected data, we selected two targets via DianaTools. For Plaur-miR1-5p, we enrolled *Emx2*, a transcription factor that plays an important role in the embryonic brain, to specify cell fates in the developing central nervous system (Bishop et al., 2002; Supplementary Table 2). For Plaur-miR1-3p, we selected *Mef2d*, a transcriptional activator that plays a critical role in neuronal apoptosis (Supplementary Table 3; Wang et al., 2009). In addition to the DianaTools predictive algorithm, we used an original approach for experimental tissue-specific detection of potential Plaur-miR1 target genes by analyzing the sequences obtained from sequencing PCR products of brain samples with primers for Plaur-miR1-5p (Figures 4C,D). We believe that this approach is more reliable than DianaTools. DianaTools is based on the algorithm, which allows target prediction with the highest score. However, this tool has disadvantages, specifically the lack of extensive experimentally validated miRNA–gene interaction datasets, forcing most available implementations to rely solely on *in silico* predicted interactions (Vlachos et al., 2015). As previously mentioned, even the most advanced miRNA target prediction algorithms exhibit high false-positive rates (Vlachos and Hatzigeorgiou, 2013). Our study is in agreement with this previously published data: among Plaur-miR1-5p targets found with the sequence assistant *Snrnp200* (U5 small nuclear ribonucleoprotein) and *Nrip3* (nuclear receptor interacting protein 3) had low scores (0.48 and 0.38, respectively), suggesting these targets would be dismissed when using DianaTools alone. Surprisingly, the expression dynamics analysis of the Plaur-miR1 targets in the mouse brain (striatum) after *Plaur* induction revealed a direct correlation between an increase in *Plaur* expression and elevated *Mef2d*, *Emx2*, and *Snrnp200* gene expression (Figures 5A–C). The elevated MEF2D expression was verified at the protein level (Figure 6A). *Nrip3* mRNA expression was not significantly changed (Figure 5D).

Therefore, continuing to elaborate on the hypothesis that in addition to the classical mechanism of miRNA action relying on the suppression of target genes and occurring when a miRNA binds to the 3′-untranslated region of the target gene, there exist an activating miRNA function implemented via miRNA interaction with 3′-UTR. Evidence supporting this concept has been published by Chu and co-authors in NAR (Chu et al., 2020). In addition, the underlying mechanism that involves miRNA binding proteins Argonaute 2 (AGO-2) and Fragile-X-metal retardation related protein 1 (FXR1) has been recently (April 2022) proposed by Jame-Chenarboo and co-authors (Faezeh Jame-Chenarboo, Hoi Hei Ng, Dawn Macdonald, Lara K. Mahal. (2022). miRNA upregulate protein and glycan expression via direct activation in proliferating cells. bioRxiv 2022.04.01.486772, preprint).^11^ Therefore, the accumulated data warrant further investigation into canonical and non-canonical miRNA action.

We verified the predicted Plaur-miR1-5p binding sites in the promoter region of the selected genes (Figure 3A), as well as in the intron region putatively located in the distal enhancer regions (Figure 3B; Broughton et al., 2016). Our hypothesis has been confirmed experimentally only for *Mef2d* and *Snrnp200* in Neuro2a cells. Specifically, we altered the *Plaur* and Plaur-miR1 expression in these cells and established the expression dependence of the target genes on *Plaur* and Plaur-miR1 (Figure 7). Moreover, using Luciferase reporter assay we confirmed the direct activating function of Plaur-miR1 on the *Mef2d* expression via 3′-UTR interaction (Figure 8). Since *Nrip3* and *Emx2* expression was not detected in Neuro2a cells, the Plaur-miR1 effect on these genes was impossible to evaluate. These data suggest that the regulation of Plaur-miR1 target mRNAs occurs at the DNA level resulting in the alterations in mRNA expression. Herein, the identified targets play an important role in physiology of the nervous system in normal and pathological conditions. *Emx2* is a transcription factor that plays an essential role in specifying cell fates in the embryonic central nervous system. *Emx2* controls several biological parameters of cortical neuroblast proliferation and subsequent cell migration of postmitotic neurons in the mouse brain (Gulisano et al., 1996), as well as imparts positional identity to cortical cells in the developing neocortex (Bishop et al., 2002). Our current findings are in accordance with the previously published papers revealing that high *Plaur* expression stimulates neuronal migration to the outer layers of mouse neocortex during embryogenesis (Shmakova et al., 2021), as well as the absence of GABA neurons in *Plaur*-knockout mice (Powell et al., 2003). The most plausible explanation for these data is that uPAR functions as a morphogenic factor in the brain, realizing its action via Plaur-miR1-5p and its target *Emx2* (Gulisano et al., 1996).

Another target gene of Plaur-miR1, *Mef2d*, encodes a developmental protein that regulates large-scale gene expression programs necessary in embryogenesis and tissue architecture maintenance, including the brain, and contributes to the regulation of neurogenesis, neuronal apoptosis and differentiation (Pon and Marra, 2016; Assali et al., 2019). *Mef2d* gene disruption could be a risk factor for multiple neurodevelopmental disorders and mental illnesses, such as autism spectrum disorders, intellectual disability and schizophrenia (Assali et al., 2019). Considering that *Plaur* polymorphisms in humans are associated with cognitive disorders (Campbell et al., 2008) in mice with behavioral dysfunction and epilepsy (Powell et al., 2003), apoptosis of neuronal cells *in vitro* (Rysenkova et al., 2018), as well as *Plaur* gene knockout leads to impaired brain formation (Eagleson et al., 2011), we hypothesize that these “protective” uPAR effects on brain neurons may be implemented via Plaur-miR1 and its target *Mef2d*.

In conclusion, we identified the novel Plaur-miR1 as a functional miRNA of *Plaur* intron 3. Furthermore, we revealed that the Plaur-miR1 expression specifically controls the MEF2D expression at the mRNA and protein levels. Taking into account our previously published data and the present results we suggest a novel role for *Plaur* as a morphogenetic factor in brain development and a marker of brain disorders.

## Data Availability Statement

The datasets presented in this study can be found in online repositories. The names of the repository/repositories and accession number(s) can be found in the article/Supplementary Material.

## Author Contributions

KDR and EVS conceived and designed the experiments. KDR, EVS, PSK, TRB, EMS, AAS, DYT, OII, KVA, and MNK carried out the experiments, analyzed the data, and performed the statistical analyses. KET, KDR, and MNK performed the bioinformatics screening. KDR, EVS, and KAR contributed to the manuscript writing. MEZ, VAT, and EVS made a substantial contribution to the conception of the manuscript. All authors read and approved the final version of the manuscript.

## Conflict of Interest

The authors declare that the research was conducted in the absence of any commercial or financial relationships that could be construed as a potential conflict of interest.

## Publisher’s Note

All claims expressed in this article are solely those of the authors and do not necessarily represent those of their affiliated organizations, or those of the publisher, the editors and the reviewers. Any product that may be evaluated in this article, or claim that may be made by its manufacturer, is not guaranteed or endorsed by the publisher.

## Footnotes

1
https://www.mirbase.org/

2
https://services.healthtech.dtu.dk/service.php?Promoter-2

3
http://lifefaculty.biu.ac.il/gershon-tamar/index.php/element-description

4
https://evryrna.ibisc.univ-evry.fr/miRNAFold

5
http://unafold.rna.albany.edu/?q=DINAMelt/Quickfold

6
http://diana.imis.athena-innovation.gr/DianaTools/index.php?r=mrmicrot/index

7
www.ncbi.nlm.nih.gov/tools/primer-blast/

8
https://eu.idtdna.com/pages/tools/oligoanalyzer/

9
https://evryrna.ibisc.univ-evry.fr/miRNAFold

10
https://www.ensembl.org

11
https://doi.org/10.1101/2022.04.01.486772

## References

[B1] Agrawal N. Dasaradhi P. V. N. Mohmmed A. Malhotra P. Bhatnagar R. K. Mukherjee S. K. (2003). RNA interference: biology, mechanism, and applications. *Microbiol. Mol. Biol. Rev.* 67 657–685. 10.1128/MMBR.67.4.657-685.2003 14665679 PMC309050

[B2] Assali A. Harrington A. J. Cowan C. W. (2019). Emerging roles for MEF2 in brain development and mental disorders. *Curr. Opin. Neurobiol.* 59 49–58. 10.1016/j.conb.2019.04.008 31129473 PMC6874740

[B3] Auyeung V. C. Ulitsky I. McGeary S. E. Bartel D. P. (2013). Beyond secondary structure: primary-sequence determinants license pri-miRNA hairpins for processing. *Cell* 152 844–858. 10.1016/j.cell.2013.01.031 23415231 PMC3707628

[B4] Bishop K. M. Rubenstein J. L. R. O’Leary D. D. M. (2002). Distinct actions of Emx1. *Emx2, and Pax6 in regulating the specification of areas in the developing neocortex*. *J. Neurosci.* 22 7627–7638. 10.1523/JNEUROSCI.22-17-07627.2002 12196586 PMC6757966

[B5] Broughton J. P. Lovci M. T. Huang J. L. Yeo G. W. Pasquinelli A. E. (2016). Pairing beyond the Seed Supports MicroRNA Targeting Specificity. *Mol. Cell* 64 320–333. 10.1016/j.molcel.2016.09.004 27720646 PMC5074850

[B6] Bruneau N. Szepetowski P. (2011). The role of the urokinase receptor in epilepsy, in disorders of language, cognition, communication and behavior, and in the central nervous system. *Curr. Pharm. Des.* 17 1914–1923. 10.2174/138161211796718198 21711233

[B7] Campbell D. B. Li C. Sutcliffe J. S. Persico A. M. Levitt P. (2008). Genetic evidence implicating multiple genes in the MET receptor tyrosine kinase pathway in autism spectrum disorder. *Autism Res.* 1 159–168. 10.1002/aur.27 19360663 PMC2678909

[B8] Chu Y. Kilikevicius A. Liu J. Johnson K. C. Yokota S. Corey D. R. (2020). Argonaute binding within 3′-untranslated regions poorly predicts gene repression. *Nucleic Acids Res.* 48 7439–7453. 10.1093/nar/gkaa478 32501500 PMC7367155

[B9] Dokanehiifard S. Soltani B. M. Parsi S. Hosseini F. Javan M. Mowla S. J. (2015). Experimental verification of a conserved intronic microRNA located in the human TrkC gene with a cell type-dependent apoptotic function. *Cell. Mol. Life Sci.* 72 2613–2625. 10.1007/s00018-015-1868-4 25772499 PMC11113298

[B10] Dokanehiifard S. Yasari A. Najafi H. Jafarzadeh M. Nikkhah M. Mowla S. J. (2017). A novel microRNA located in the TrkC gene regulates the Wntsignaling pathway and is differentially expressed in colorectal cancer specimens. *J. Biol. Chem.* 292 7566–7577. 10.1074/jbc.M116.760710 28100780 PMC5418054

[B11] Eagleson K. L. Campbell D. B. Thompson B. L. Bergman M. Y. Levitt P. (2011). The autism risk genes MET and PLAUR differentially impact cortical development. *Autism Res.* 4 68–83. 10.1002/aur.172 21328570 PMC3644181

[B12] Eden G. Archinti M. Furlan F. Murphy R. Degryse B. (2011). The urokinase receptor interactome. *Curr. Pharm. Des.* 17 1874–1889. 10.2174/138161211796718215 21711237

[B13] Gkirtzou K. Tsamardinos I. Tsakalides P. Poirazi P. (2010). MatureBayes: a probabilistic algorithm for identifying the mature miRNA within novel precursors. *PLoS One* 5:e11843. 10.1371/journal.pone.0011843 20700506 PMC2917354

[B14] Gulisano M. Broccoli V. Pardini C. Boncinelli E. (1996). Emx1 and Emx2 show different patterns of expression during proliferation and differentiation of the developing cerebral cortex in the mouse. *Eur. J. Neurosci.* 8 1037–1050. 10.1111/j.1460-9568.1996.tb01590.x 8743751

[B15] Kjaergaard M. Hansen L. V. Jacobsen B. Gardsvoll H. Ploug M. (2008). Structure and ligand interactions of the urokinase receptor (uPAR). *Front. Biosci.* 13:5441–5461. 10.2741/3092 18508598

[B16] Klimovich P. S. Semina E. V. Karagyaur M. N. Rysenkova K. D. Sysoeva V. Y. Mironov N. A. (2020). Urokinase receptor regulates nerve regeneration through its interaction with α5β1-integrin. *Biomed. Pharmacother.* 125:110008. 10.1016/j.biopha.2020.110008 32187956

[B17] Lee D. Shin C. (2018). Emerging roles of DROSHA beyond primary microRNA processing. *RNA Biol.* 15 186–193. 10.1080/15476286.2017.1405210 29171328 PMC5798959

[B18] Mahmood N. Mihalcioiu C. Rabbani S. A. (2018). Multifaceted Role of the Urokinase-Type Plasminogen Activator (uPA) and Its Receptor (uPAR): diagnostic. *Prognos, Therap. Appl. Front. Oncol.* 8:24. 10.3389/fonc.2018.00024 29484286 PMC5816037

[B19] Ørom U. A. Nielsen F. C. Lund A. H. (2008). MicroRNA-10a binds the 5′UTR of ribosomal protein mRNAs and enhances their translation. *Mol. Cell* 30 460–471. 10.1016/j.molcel.2008.05.001 18498749

[B20] Paraskevopoulou M. D. Georgakilas G. Kostoulas N. Vlachos I. S. Vergoulis T. Reczko M. (2013). DIANA-microT web server v5.0: service integration into miRNA functional analysis workflows. *Nucleic Acids Res.* 41 W169–W173. 10.1093/nar/gkt393 23680784 PMC3692048

[B21] Parfenova E. V. Plekhanova O. S. Men’shikov M. I. Stepanova V. V. Tkachuk V. A. (2009). [Regulation of growth and remodeling of blood vessels: the unique role of urokinase]. *Ross. Fiziol. zhurnalIm. I.M. Sechenova* 95 442–464. 19569522

[B22] Place R. F. Li L.-C. Pookot D. Noonan E. J. Dahiya R. (2008). MicroRNA-373 induces expression of genes with complementary promoter sequences. *Proc. Natl. Acad. Sci U. S A* 105 1608–1613. 10.1073/pnas.0707594105 18227514 PMC2234192

[B23] Pon J. R. Marra M. A. (2016). MEF2 transcription factors: developmental regulators and emerging cancer genes. *Oncotarget* 7 2297–2312. 10.18632/oncotarget.6223 26506234 PMC4823036

[B24] Powell E. M. Campbell D. B. Stanwood G. D. Davis C. Noebels J. L. Levitt P. (2003). Genetic disruption of cortical interneuron development causes region- and GABA cell type-specific deficits, epilepsy, and behavioral dysfunction. *J. Neurosci.* 23 622–631. 10.1523/JNEUROSCI.23-02-00622.2003 12533622 PMC6741866

[B25] Reczko M. Maragkakis M. Alexiou P. Grosse I. Hatzigeorgiou A. G. (2012). Functional microRNA targets in protein coding sequences. *Bioinformatics* 28 771–776. 10.1093/bioinformatics/bts043 22285563

[B26] Rysenkova K. D. Klimovich P. S. Shmakova A. A. Karagyaur M. N. Ivanova K. A. Aleksandrushkina N. A. (2020). Urokinase receptor deficiency results in EGFR-mediated failure to transmit signals for cell survival and neurite formation in mouse neuroblastoma cells. *Cell Signal* 75:109741. 10.1016/j.cellsig.2020.109741 32822758

[B27] Rysenkova K. D. Semina E. V. Karagyaur M. N. Shmakova A. A. Dyikanov D. T. Vasiluev P. A. (2018). CRISPR/Cas9 nickase mediated targeting of urokinase receptor gene inhibits neuroblastoma cell proliferation. *Oncotarget* 9 29414–29430. 10.18632/oncotarget.25647 30034627 PMC6047682

[B28] Semina E. V. Rubina K. A. Shmakova A. A. Rysenkova K. D. Klimovich P. S. Aleksanrushkina N. A. (2020). Downregulation of uPAR promotes urokinase translocation into the nucleus and epithelial to mesenchymal transition in neuroblastoma. *J. Cell. Physiol.* 235 6268–6286. 10.1002/jcp.29555 31990070 PMC7318179

[B29] Semina E. V. Rubina K. A. Sysoeva V. Y. Makarevich P. I. Parfyonova Y. V. Tkachuk V. A. (2016). The role of urokinase in vascular cell migration and in regulation of growth and branching of capillaries. *Cell tissue biol.* 10 37–46. 10.1134/S1990519X16010089 26863767

[B30] Shmakova A. A. Balatskiy A. V. Kulebyakina M. A. Schaub T. Karagyaur M. N. Kulebyakin K. Y. (2021). Urokinase Receptor uPAR Overexpression in Mouse Brain Stimulates the Migration of Neurons into the Cortex during Embryogenesis. *Russ. J. Dev. Biol.* 52 53–63. 10.1134/S1062360421010069

[B31] Shmakova A. A. Rubina K. A. Rysenkova K. D. Gruzdeva A. M. Ivashkina O. I. Anokhin K. V. (2020). Urokinase receptor and tissue plasminogen activator as immediate-early genes in pentylenetetrazole-induced seizures in the mouse brain. *Eur. J. Neurosci.* 51 1559–1572. 10.1111/ejn.14584 31587391

[B32] Sloutskin A. Danino Y. M. Orenstein Y. Zehavi Y. Doniger T. Shamir R. (2015). ElemeNT: a computational tool for detecting core promoter elements. *Transcription* 6 41–50. 10.1080/21541264.2015.1067286 26226151 PMC4581360

[B33] Tav C. Tempel S. Poligny L. Tahi F. (2016). miRNAFold: a web server for fast miRNA precursor prediction in genomes. *Nucleic Acids Res.* 44 W181–W184. 10.1093/nar/gkw459 27242364 PMC4987958

[B34] Tkachuk V. A. Parfyonova Y. V. Plekhanova O. S. Stepanova V. V. Menshikov M. Y. Semina E. V. (2019). [Fibrinolytics: from the thrombolysis to the processes of blood vessels growth and remodeling, neurogenesis, carcinogenesis and fibrosis]. *Ter. Arkh.* 91 4–9. 10.26442/00403660.2019.09.000411 32598807

[B35] Tkachuk V. A. Plekhanova O. S. Parfyonova Y. V. (2009). Regulation of arterial remodeling and angiogenesis by urokinase-type plasminogen activator. *Can. J. Physiol. Pharmacol.* 87 231–251. 10.1139/Y08-113 19370078

[B36] Vlachos I. S. Hatzigeorgiou A. G. (2013). Online resources for miRNA analysis. *Clin. Biochem.* 46 879–900. 10.1016/j.clinbiochem.2013.03.006 23518312

[B37] Vlachos I. S. Zagganas K. Paraskevopoulou M. D. Georgakilas G. Karagkouni D. Vergoulis T. (2015). DIANA-miRPath v3.0: deciphering microRNA function with experimental support. *Nucleic Acids Res.* 43 W460–W466. 10.1093/nar/gkv403 25977294 PMC4489228

[B38] Wang X. She H. Mao Z. (2009). Phosphorylation of neuronal survival factor MEF2D by glycogen synthase kinase 3 beta in neuronal apoptosis. *J. Biol. Chem.* 284 32619–32626. 10.1074/jbc.M109.067785 19801631 PMC2781676

[B39] Xue C. Li F. He T. Liu G.-P. Li Y. Zhang X. (2005). Classification of real and pseudo microRNA precursors using local structure-sequence features and support vector machine. *BMC Bioinform.* 6:310. 10.1186/1471-2105-6-310 16381612 PMC1360673

[B40] Yepes M. Woo Y. Martin-Jimenez C. (2021). Plasminogen Activators in Neurovascular and Neurodegenerative Disorders. *Int. J. Mol. Sci* 22:4380. 10.3390/ijms22094380 33922229 PMC8122722

