# Supplementary material for: Identification of a Novel Small RNA Encoded in the Mouse Urokinase Receptor uPAR Gene (Plaur) and Its Molecular Target Mef2d
Source: Front Mol Neurosci. 2022 Jul 6;15:865858. doi: 10.3389/fnmol.2022.865858 (PMC9298986; doi:10.3389/fnmol.2022.865858)
Supplement: Supplementary file 1 [file Data_Sheet_1.docx]

***Supplementary Material***

**А**


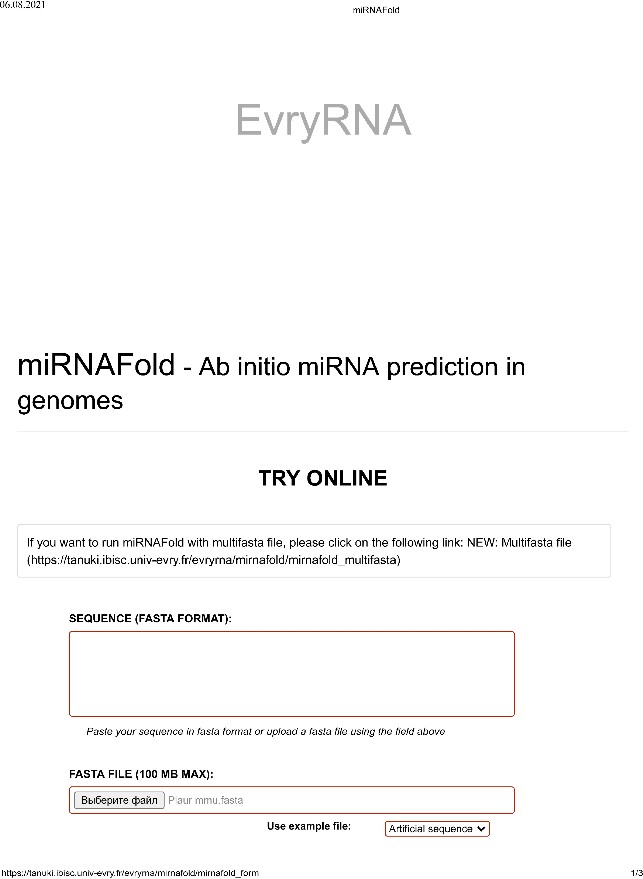

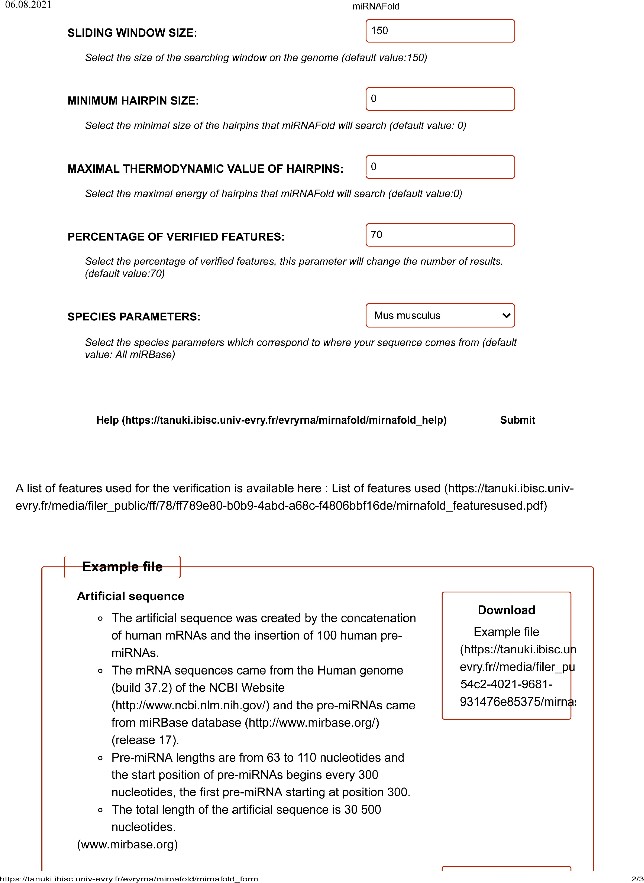


**В**


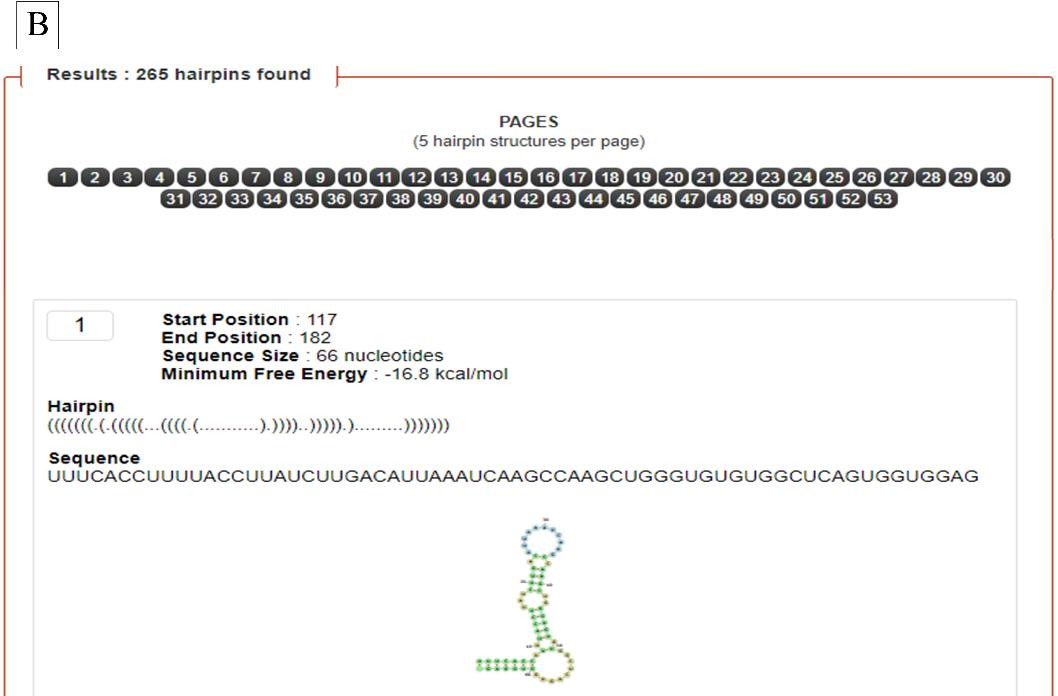


**Supplementary Figure S1**. Prediction of hairpin structures located in *Plaur*. **(A)** A screenshot of the miRNAFold program windows and the parameters used for the search. **(B)** The result of the search in the miRNAFold program was 256 hairpin structures, from which three stem-loop structures were further selected following their internal localisation and overall stability (≤-15 kJ/mol). Reproduced with permission from (Prof. Fariza Tahi), available at (<https://evryrna.ibisc.univ-evry.fr/miRNAFold>).


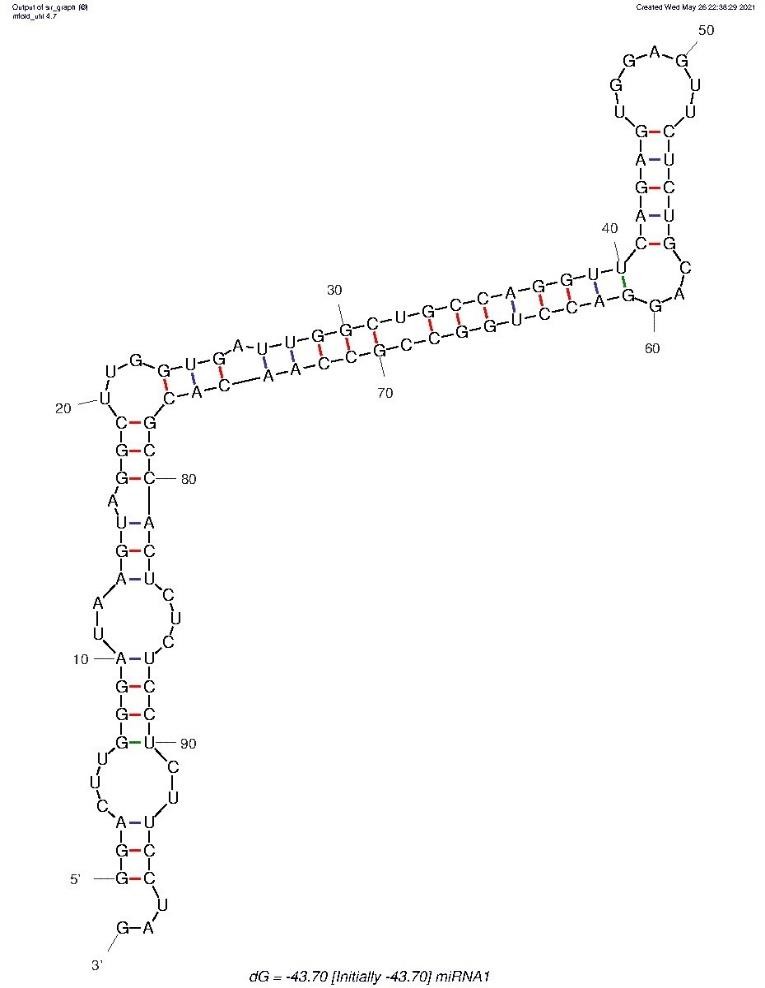


**Supplementary Figure S2.** Plaur-pre-miR1 secondary structure predicted by Quickfold.


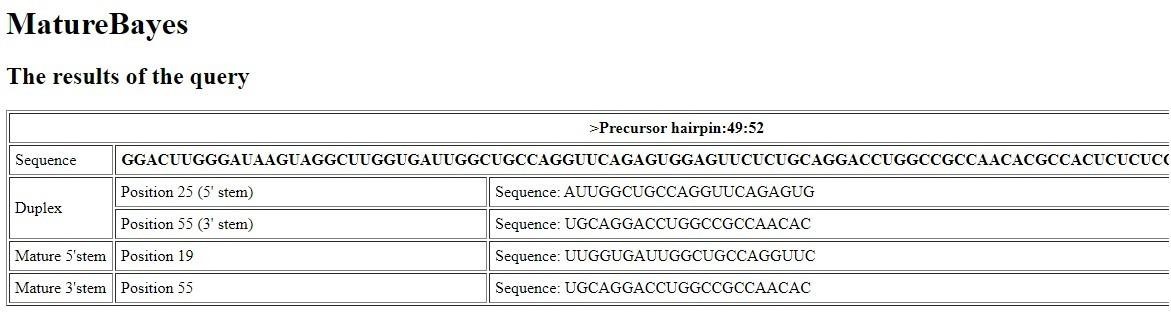


**Supplementary Figure S3.** The results of Plaur-premiR1 processing by the Drosha enzyme using the MatureBayes program. Reproduced with permission from (Dr. Yiota Poirazi), available at (<http://unafold.rna.albany.edu/?q=DINAMelt/Quickfold>).


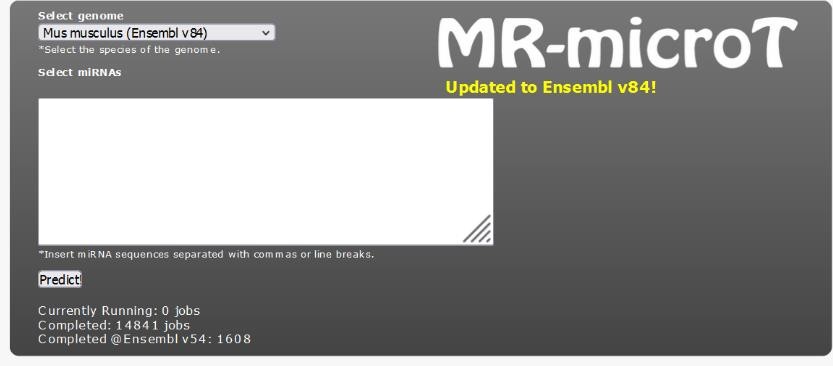
**A**

**B**

Results of the search for target genes Plaur-miR-5p


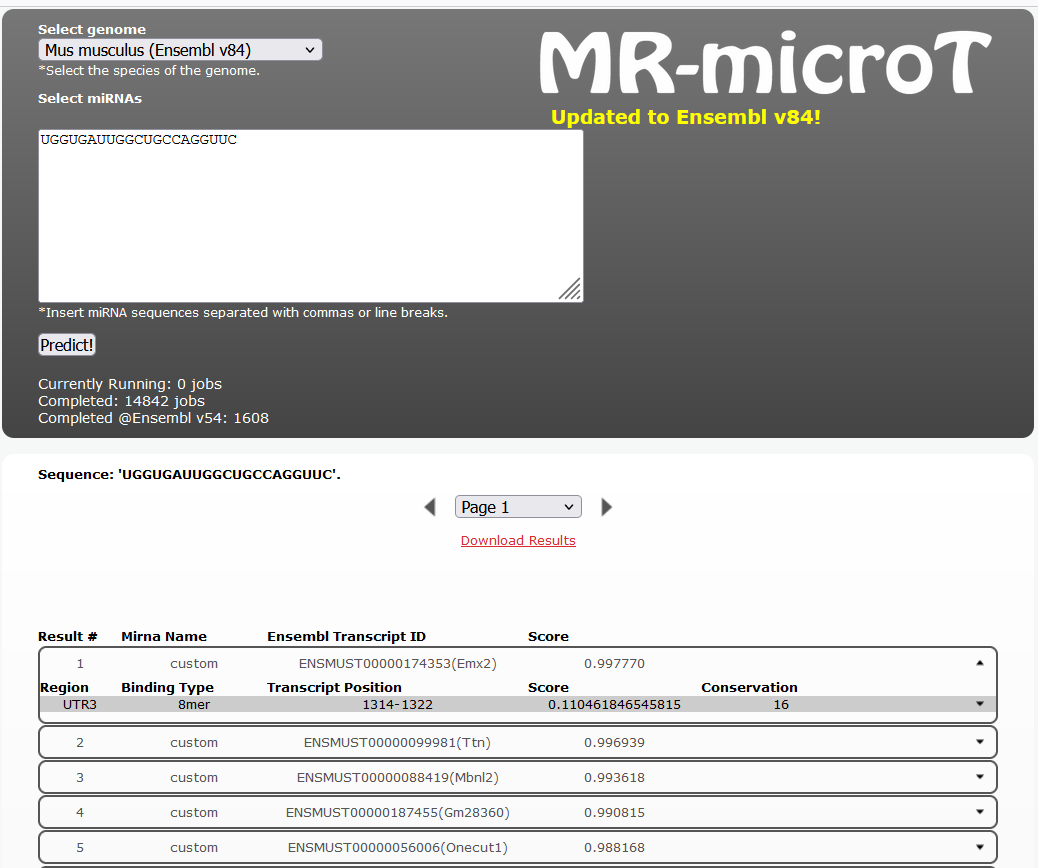


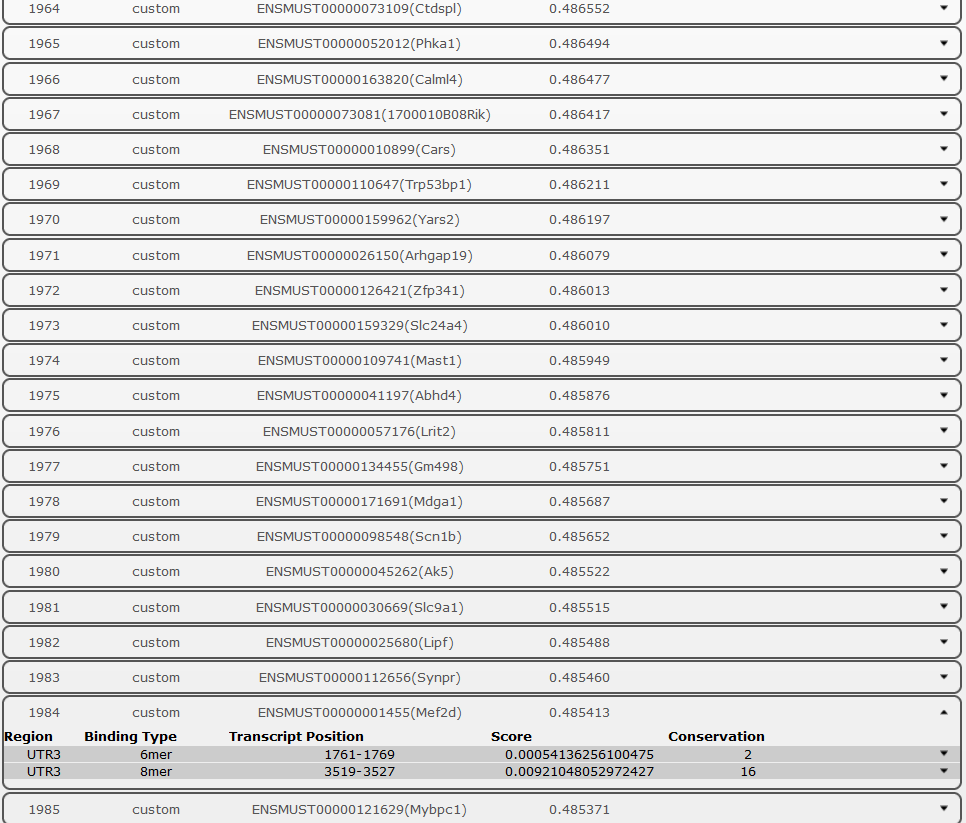


Results of the search for target genes Plaur-miR-3p


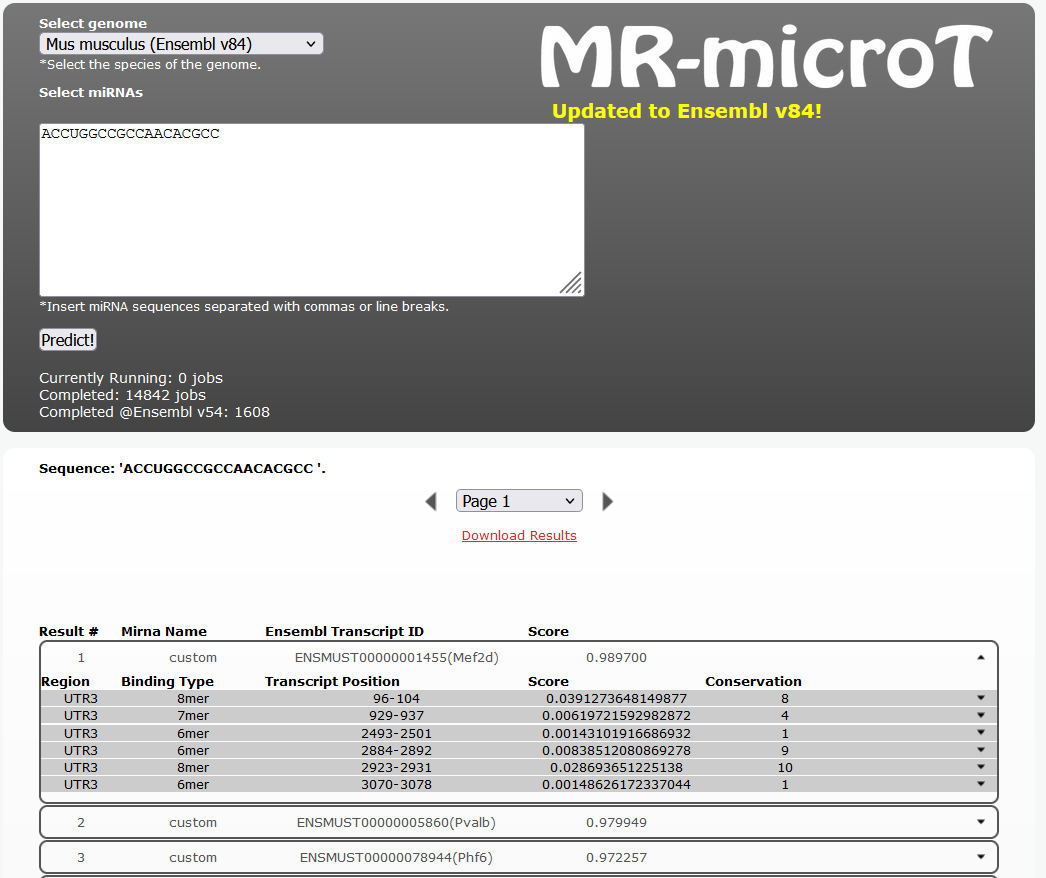


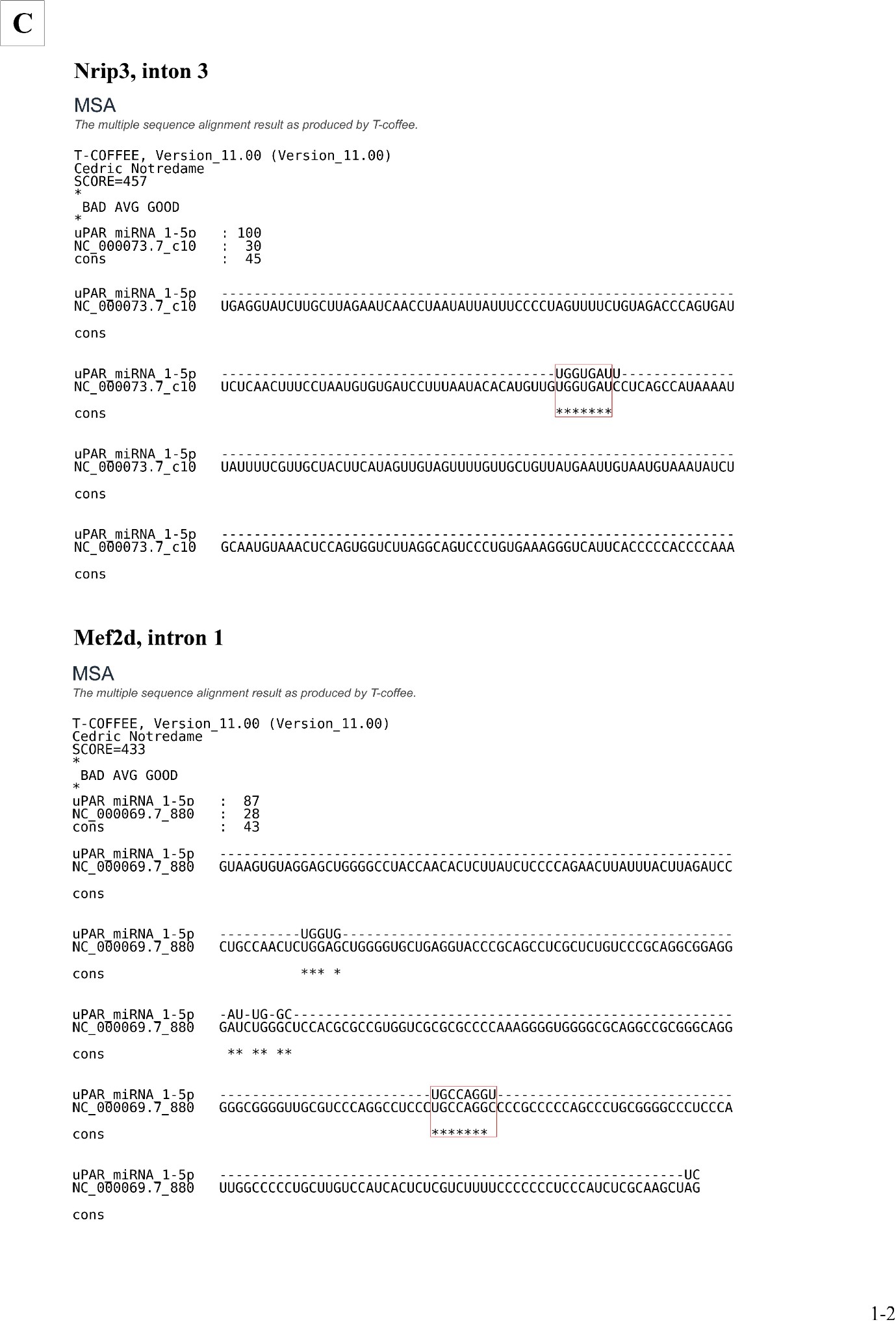

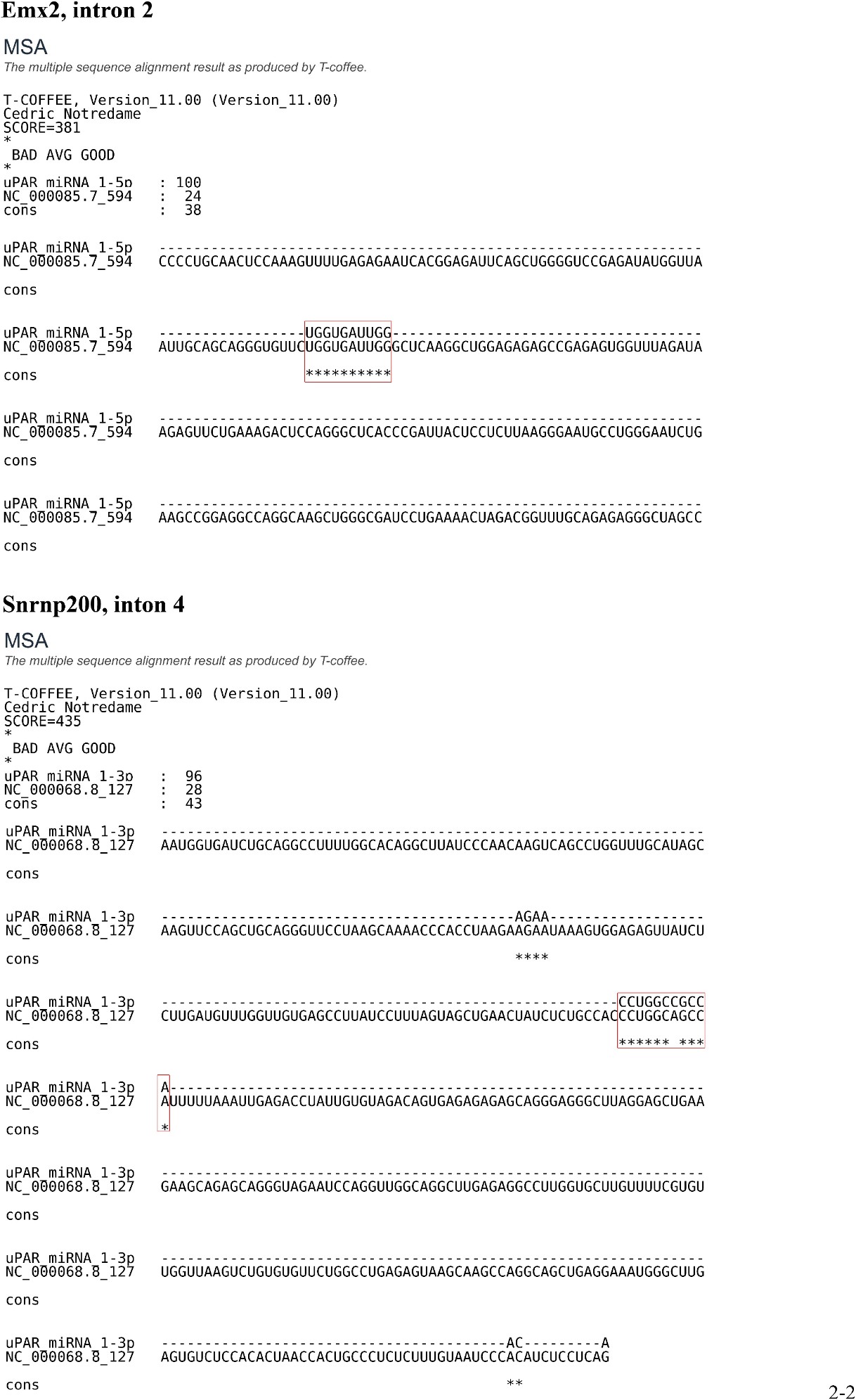


**Supplementary Figure S4.** Target gene prediction for Plaur-miR1-3p and Plaur-miR1-5p and intron binding sites for Plaur-miR1-3p and Plaur-miR1-5p within the selected genes *Mef2d* and *Emx2*. **(A)** A screenshot from the DianaTools MR-microT web service ([ttps://mrmicrot.imsi.athenarc.gr/?r=mrmicrot/](https://mrmicrot.imsi.athenarc.gr/?r=mrmicrot/)) for Plaur-miR1-3p and Plaur-miR1-5p target gene searching. *Mus musculus* genome (Ensemblv84) was used for target mRNA prediction (Reczko et al., 2021; Kanellos et al., 2014). **(B)** A screenshot from the DianaTools MR-microT web service with target genes prediction for Plaur-miR1-3p and Plaur- miR1-5p. Predicted mRNA targets with the corresponding binding score are shown. **(C)** The results of the alignment of mature uPAR-miR1 forms and introns of the selected genes – *Nrip3* (intron3), *Snrnp200* (intron4), *Emx2* (intron2), *Mef2d* (intron1) – was performed by using the M-Coffee tool of the T-COFFEE Multiple Sequence Alignment Serverweb service. Reproduced with permission from (Dr. Cedric Notredame).


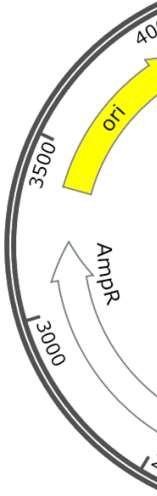

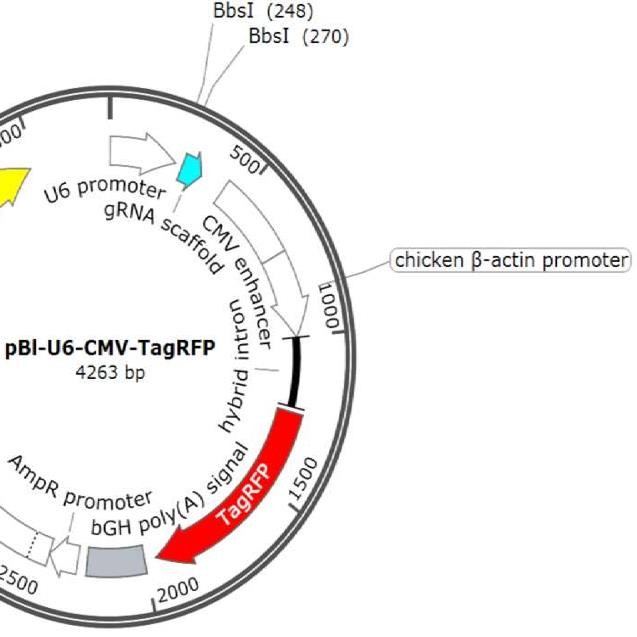


**Supplementary Figure S5.** Vector map of pBl-U6-CMV-RFP.


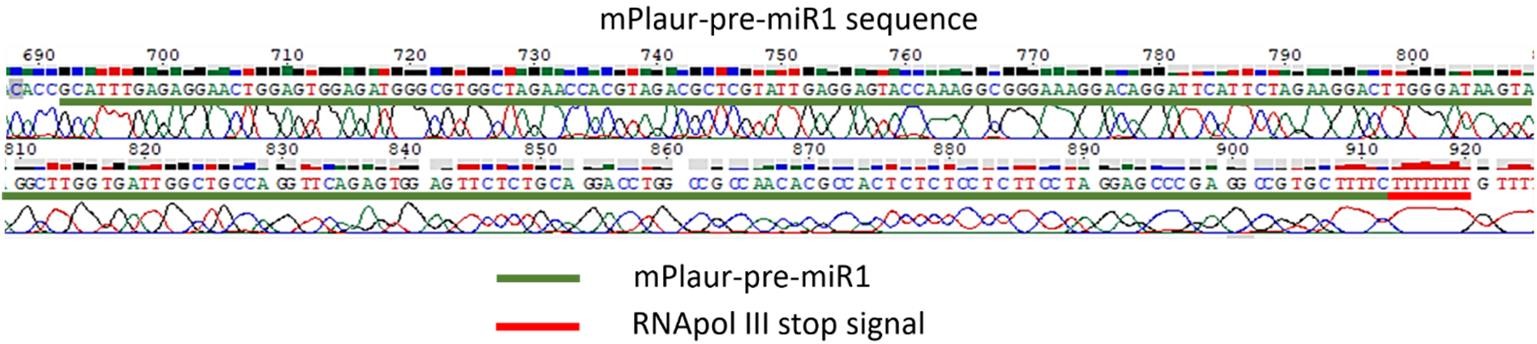


**Supplementary Figure S6.** The sequence of a part of the pBl-U6-CMV-RFP vector, encoding Plaur-pre-miR1 complementary DNA (cDNA). The sequencing was performed by Evrogen (Moscow, Russia) using u6 Primer 5′-CCTATTTCCCATGATTCCTTCATATTTGC-3′.


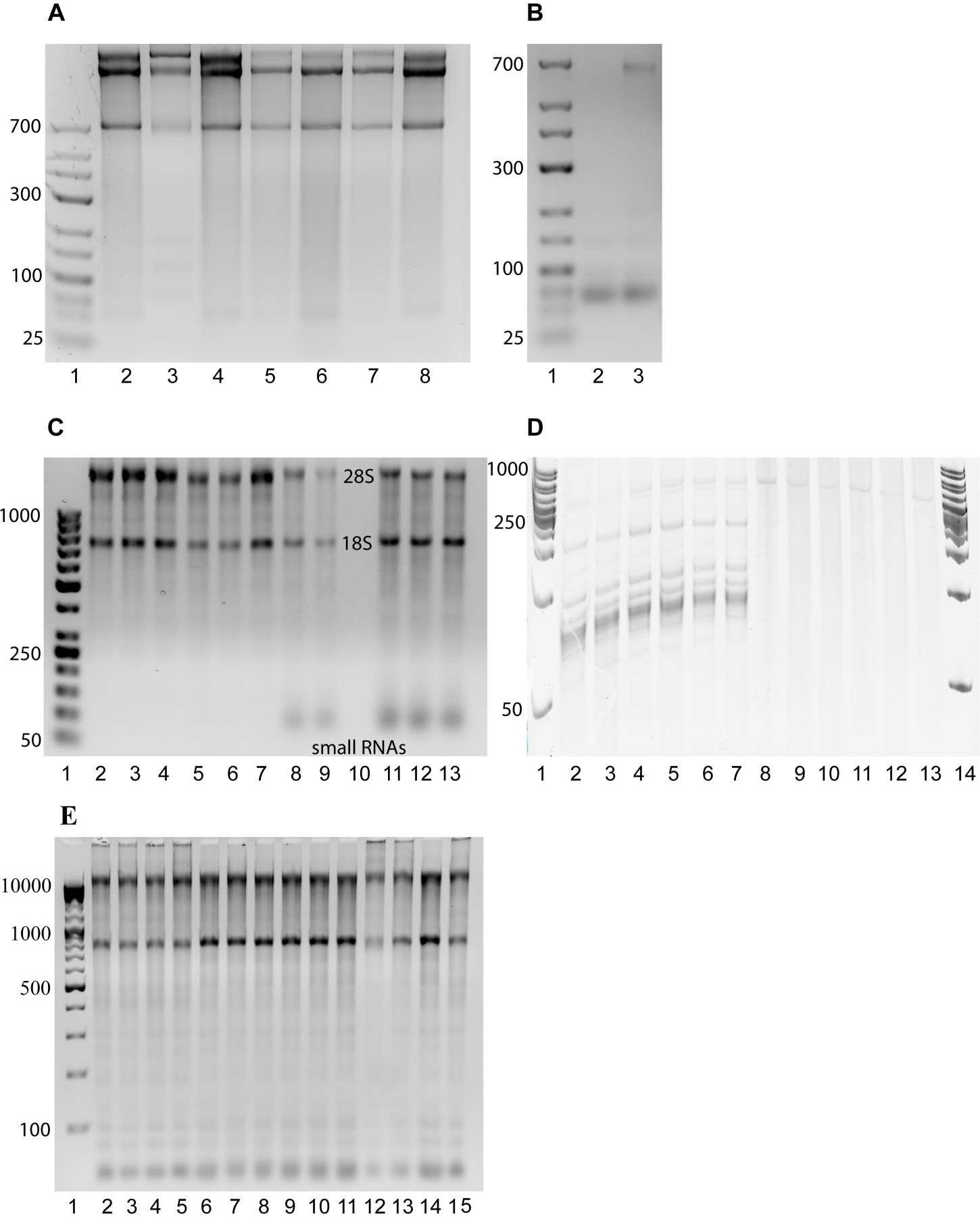


**Supplementary Figure S7.** RNA quality assessment. (**A**) Electrophoresis of total RNA isolated from Neuro2a cells and separated in a 1% agarose gel. Lanes: 1 – DNA lader;2 and 3 – Neuro2a; 4 and 5 – Neuro2a-Plaur-miR1; 6 – Neuro2a KO uPAR; 7 and 8 – Neuro2a KO uPAR Plaur-miR1. (**B**) Electrophoresis of small RNA isolated from Neuro2a cells and separated in a 1% agarose gel 1%. Lanes: 1–DNA ladder; 2 – Neuro2a small RNA; 3 – Neuro2aKO uPAR small RNA. (**C**) Electrophoresis of total RNA and large RNA fraction isolated from the posterior cortex and separated in a 1% agarose gel. Lanes:1–DNA ladder (Cleaver Scientific, Cat. No. CSL-MDNA-BR); 2–4 – primary cortex 0h, large RNA fraction; 5–7 – primary cortex 3h, large RNA fraction; 8–10 – primary cortex 0h, total RNA; 11–13 – primary cortex 3h, total RNA. (**D**) Electrophoresis of small and large RNA fraction isolated from posterior cortex and separated in a 12% PAAG with 6M urea. Lanes: 1 – DNA ladder (Cleaver Scientific, Cat. No. CSL-MDNA-BR); 2–4 – 0h, small RNA fraction; 5–7 – 3h, small RNA fraction; 8–10 – 0h, large RNA fraction; 11–13 – 3h large RNA fraction; 14 –DNA ladder. (**E**) Electrophoresis of total RNA isolated from the striatum and separated in a 1% agarose gel. Lanes: 1 – Gene Ruler DNA Ladder Mix (#SM0333); 2–3 – 0h; 4 and 5 –30 min; 6 and 7 – 1h; 8 and 9 – 3h; 10 and 11 – 6h; 12 and 13 – 24h; 14 and 15 – 72h.


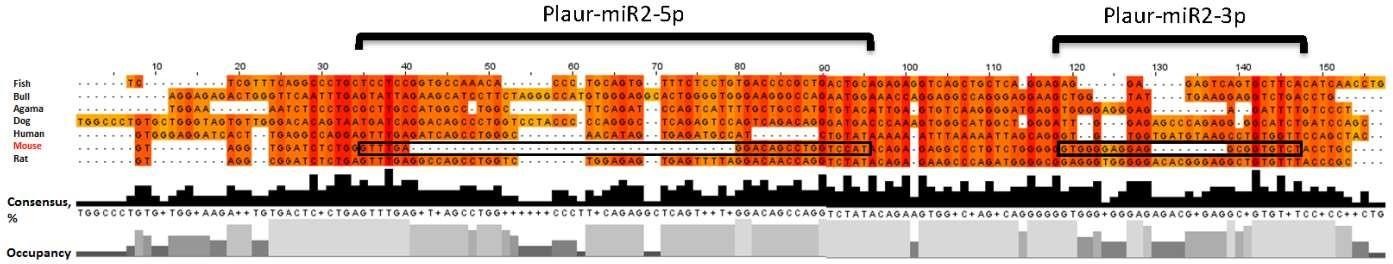


**A**

**B**

**Supplementary Figure S8.** Identification of novel miRNAs in the *Mus musculus Plaur* gene. The intronic region corresponding to the novel miRNAs Plaur-miR2-3p and Plaur-miR2-5p (**A**) as well as Plaur-miR3-3p and Plaur-miR3-5p (**B**) in the *Plaur* gene is highly conserved among vertebrates. Multiple sequence alignment with ClustalOmega (visualisation with Jalview 2.11.0) revealed consensus regions matched to mature Plaur-miR2-3p and Plaur-miR3-5p. Areas corresponding to the sequences of mature miRNAs are circled in frames; vertebrate species are indicated on the right. The consensus diagram shows the occurrence of one nucleotide in a given position. The occupancy diagram shows the number of nucleotides in a given position.


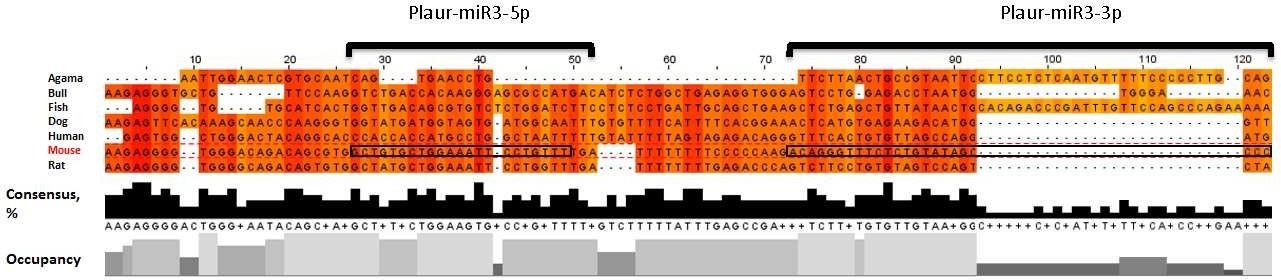

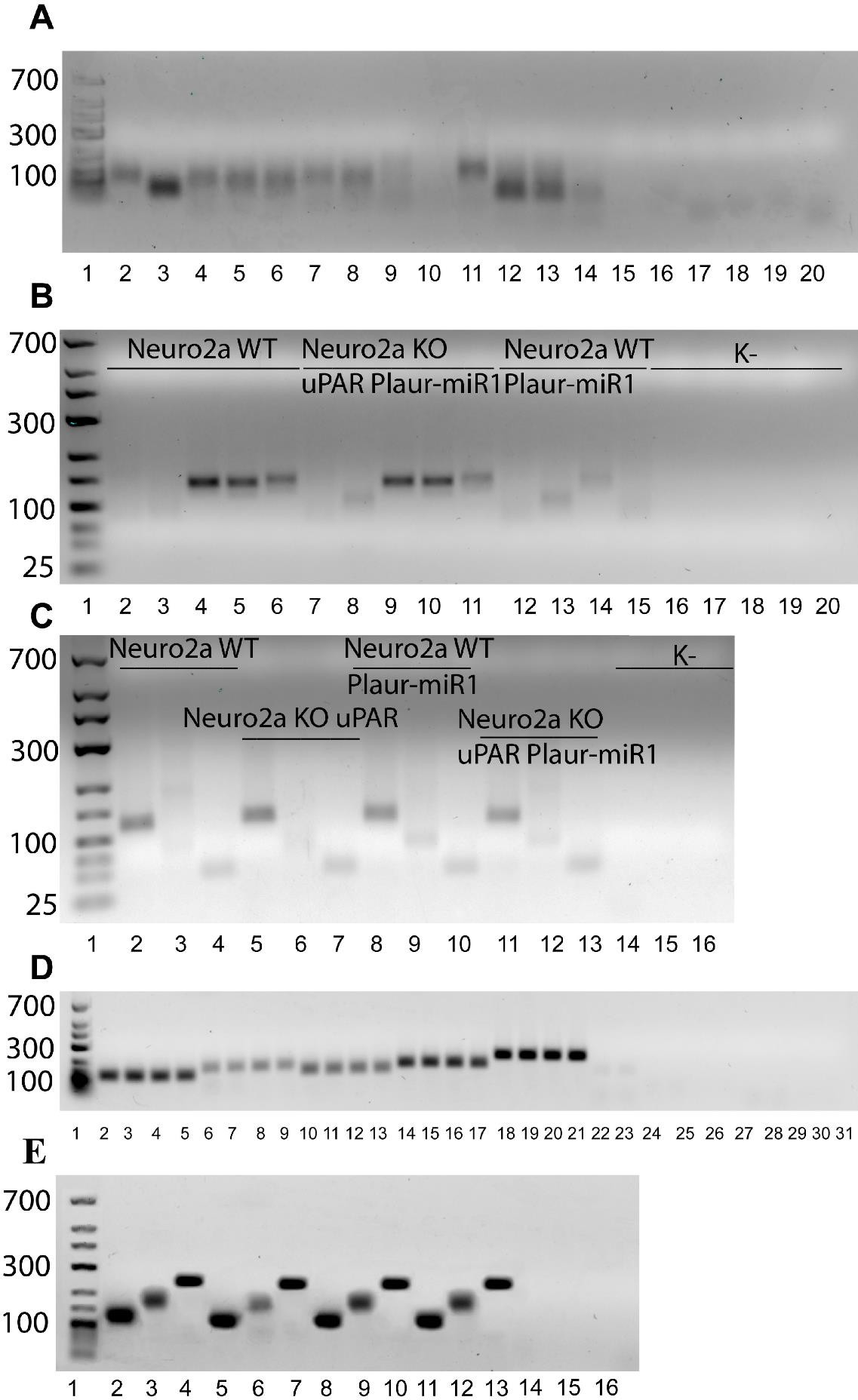


**Supplementary Figure S9.** Quality assessment of quantitative real-time polymerase chain reaction (qPCR) products. (**A**) Electrophoresis of mature Plaur-miR1 and Plaur-miR3 from mouse posterior cortex small RNA fraction. Lanes: 1 – DNA ladder; 2 – Plaur-miR1-3p; 3 – Plaur-miR1-5p; 4–6 – Plaur-miR3-3p; 7–10 – Plaur-miR3-5p; 11 – *Snord68*; 12–14 – *Snord95*; 15–20 – negative controls with primers for Plaur-miR1-3p, Plaur-miR1-5p, Plaur- miR3-3p, Plaur-miR3-5p, *Snord68* and *Snord95*, respectively. (**B**) Electrophoresis of mature Plaur-miR1 from Neuro2a small RNA fraction. Lanes: 1 – DNA ladder; 2, 7, 12 and 16 – Plaur- miR1-3p; 3, 8, 13 and 17 – Plaur-miR1-5p; 4, 9 and 18 – *Snord68*; 5, 10 and 19 – *Snord95*; 6, 11, 14 and 20 – *Snord61*. (**C**) Electrophoresis of mature Plaur-miR1 from Neuro2a small RNA fraction. Lanes: 1 – DNA ladder; 2, 5, 8 and 11 – *Snord95*; 3, 6, 9 and 12 – Plaur- miR1-3p; 4, 7, 10 and 13 – Plaur-miR1-5p. (**D**) Electrophoresis of target gene *Nrip3*, *Snrnp200*, *Emx2* and *Mef2d* mRNA from mouse striatum total RNA fraction. To verify the production of the single gene-specific product in each sample, we performed agarose gel electrophoresis after qPCR. Lanes: 1 – DNA ladder, low range; 2–5, 22 and 23 – *Actb* (112 bp); 6–9, 24 and 25 – *Nrip3* (149bp); 10–13,26 and 27–*Snrnp200* (123bp); 14–17, 28 and

29 – *Emx2* (154bp); 18–21, 30 and 31 – *Mef2d* (198bp). Lanes 2, 3, 6, 7, 10, 11, 14, 15, 18

and 19 contain primers and DNA template from the striatum of control mice; lanes 4, 5, 8, 9, 12, 13, 16, 17, 20 and 21 contain primers and DNA template from mouse striatum 3 h after *Plaur* induction; lanes 22–31 are negative controls that contain primers without DNA template. (**E**) Electrophoresis of target gene *Snrnp200* and *Mef2d* mRNA from Neuro2a total RNA fraction. Lanes: 1 – DNA ladder, low range; 2, 5, 8, 11 and 14 – *Actb* (112 bp); 3, 6, 9, 12 and 15 – *Snrnp200* (123 bp); 4, 7, 10, 13 and 16 – *Mef2d* (198bp). Lanes 2–4 contain primers and DNA template from Neuro2a wild type; lanes 5–7 contain primers and DNA template from Neuro2a with knockout of uPAR; lanes 8–10 contain primers and DNA template from Neuro2a wild type with hyperexpression of Plaur-miR1; lanes 11–13 contain primers and DNA template from Neuro2a with knockout of uPAR and hyperexpression of Plaur-miR1; lanes 14–16 are negative controls that contain primers without DNA template.


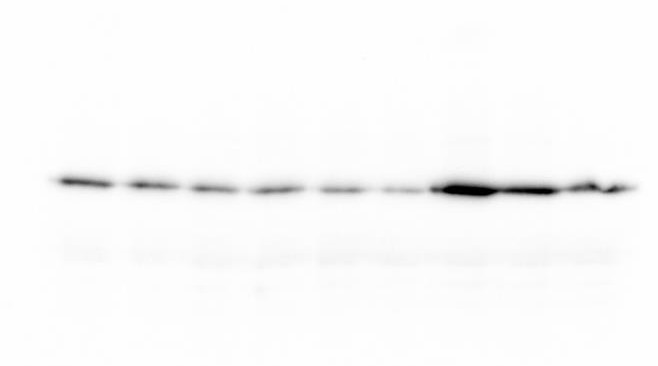


Figure 6A, MEF2D


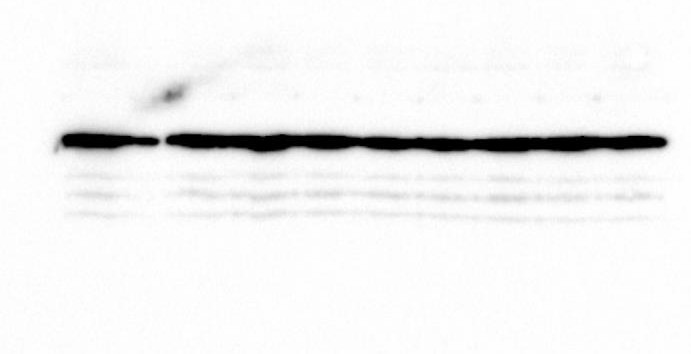


Figure 6A, β-actin


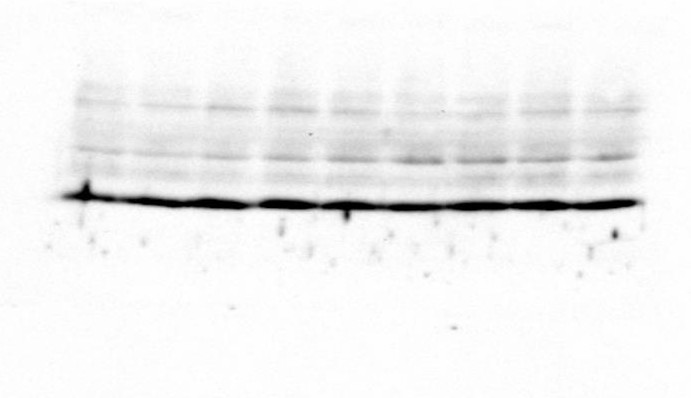


Figure 6B, EMX2


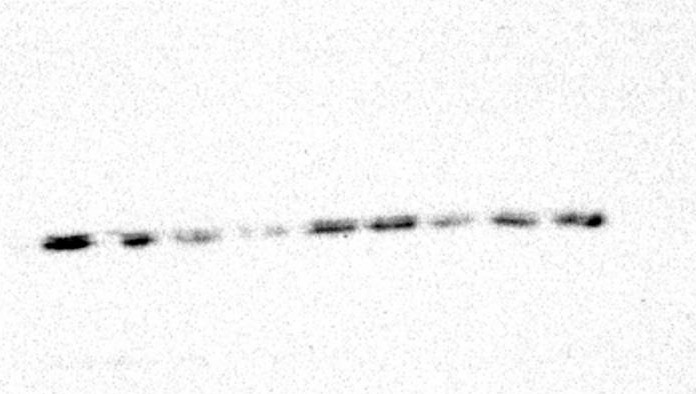


Figure 6B, β-actin


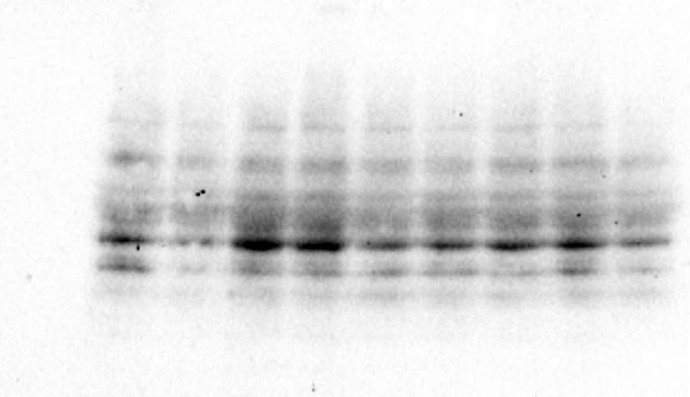


Figure 6C, SNRNP200


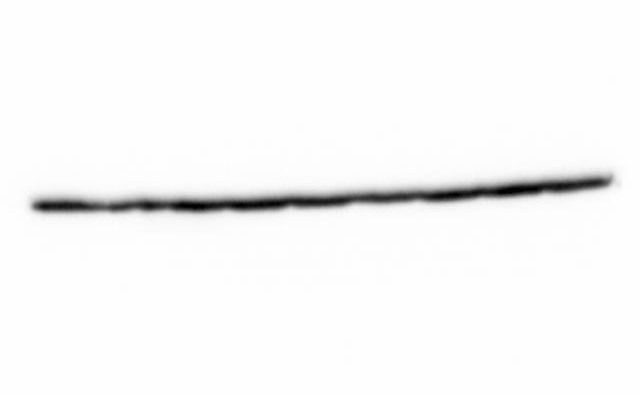


Figure 6C, β-actin

**Supplementary figure S10**. Original uncropped western blot images of Plaur-miR1 target proteins expression MEF2D, EMX2 and SNRNP200; β-actin is a protein loading control.

# Emx2 3′-UTR


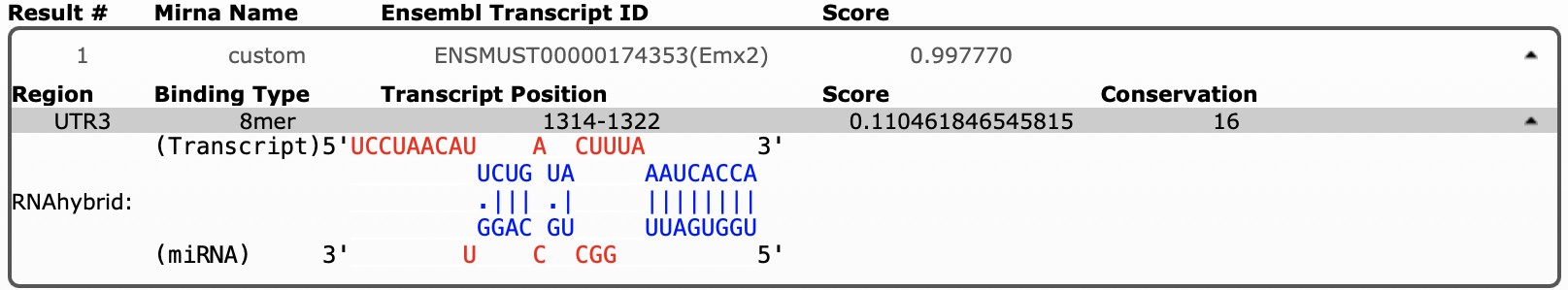


1.
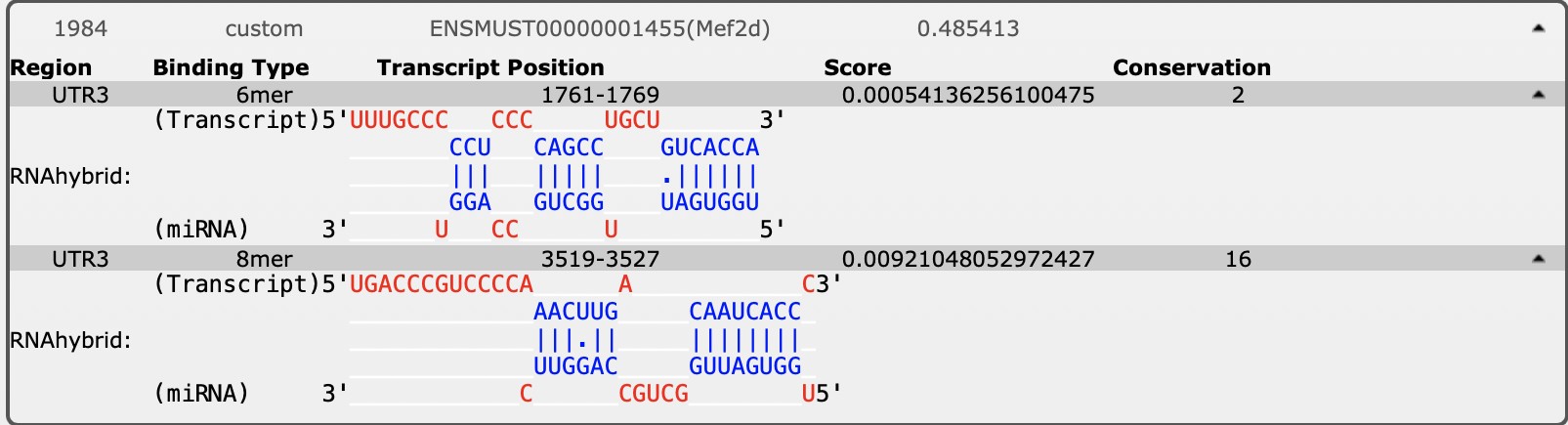
**Mef2d 3′-UTRs, 2 sequences**

# Snrnp200, CDS


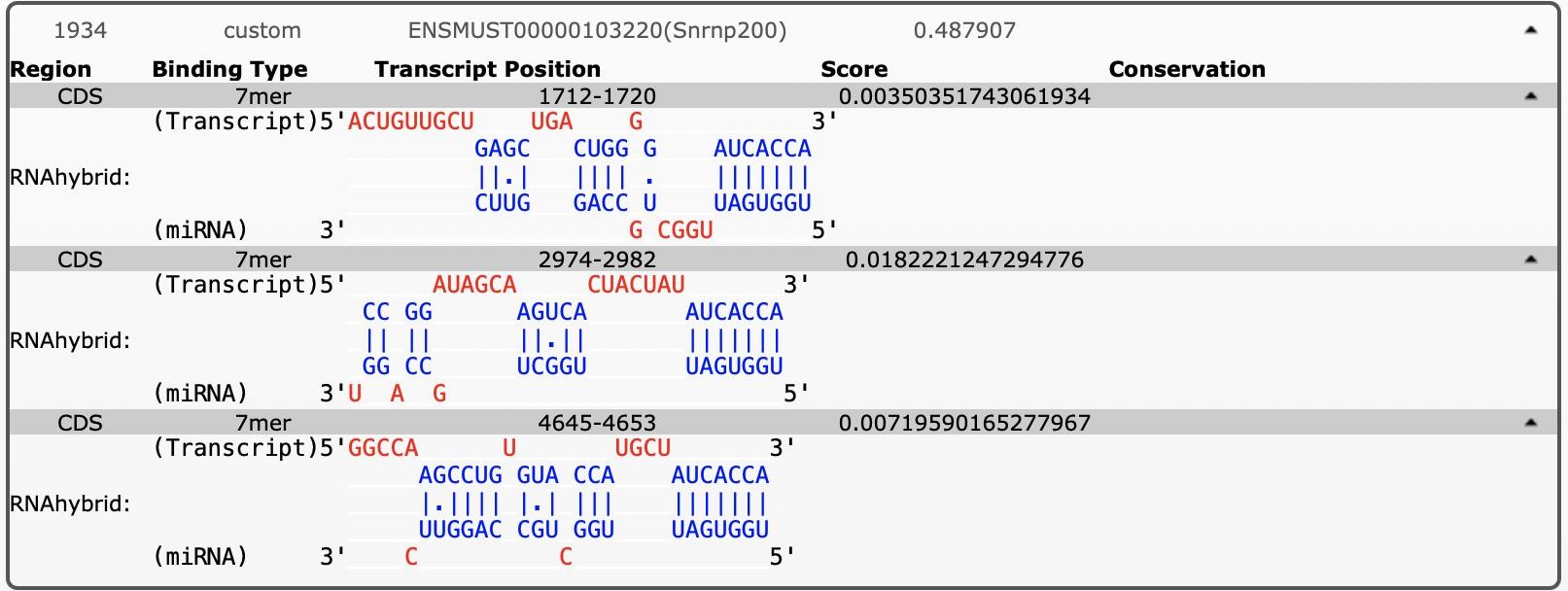


**Supplementary figure S11.** Oligo Duplexes of the Plaur-miR1 and target genes. (1), (2) – shows the binding sites for miR1-5p to 3′-UTR of Emx2 and 3′-UTR of Mef2d. (3) - shows the binding site for miR1-5p to CDS of Snrnp200. The sequences including these regions were cloned into 3′-UTR or CDS of the luciferase gene containing plasmid PGl-3. mRNA-miRNA bound and unbound nucleotides are highlighted in blue and red, respectively.

**Supplementary Table S1.** List of the primers used in the study.

| **Primer name** | **Primer sequence (5′→3′)** | **Amplicon size (base pairs)** |
| --- | --- | --- |
| *Plaur* | Forward CGCCACAAACCTCTGCAAC  Reverse CTCTGTAGGATAGCGGCATTG | 151 |
| Plaur-miR1-3p | Forward AGAACCTGGCCGCCAACA | ≈100 |
| Plaur-miR1-5p | Forward TGGTGATTGGCTGCCAGGTTC | ≈100 |
| SNORD61 | Forward AACACGCGCTGTGATGAATTTGA | ≈100–150 |
| SNORD68 | Forward AACACGCTGATGACATTCTCCG | ≈100–150 |
| SNORD95 | Forward AACACGTGCCAACAGTGATGAC | ≈100–150 |
| 10x miScript  Universal Primer | Reverse sequence is confidential manufacturing  information (miScript SYBR® Green PCRK it (#218073) |  |
| *Nrip3* | Forward AGGAGACAGACATGCGGGAA  Reverse GTTATGGGGTTGCGTGTCCT | 149 |
| *Snrnp200* | Forward TGATCGCTGGCTCTCTTGTG  Reverse CTCAGAGCAGACACAGGCAA | 123 |
| *Emx2* | Forward GTCATCGCTTCCAAGGGAAC  Reverse  GCTCCCACCACGTAATGGTT | 154 |
| *Mef2d* | Forward GTACAACGAGCCACACGAGA  Reverse  GCCGGAACAGATGACCCATA | 198 |
| Actb | Forward AGTGTGACGTTGACATCCGTA  Reverse  GCCAGAGCAGTAATCTCCTTCT | 112  (Semina et al., 2016) |
| Primers used for cloning in Luciferase reporter assay | | |
| Emx2 3′-UTR 2191-2281 (NM_010132.2) | Forward GGGAGTCTAGATTAATTCTATTTCTT GGATATTTCCTTTCCTAACAT  Reverse GGGAGGGCCGGCCCAATCTCTCCAA  CCACTAAAAGGAAAAGT | 114 |

| Mef2d 3′-UTR 3376-3499 (NM_001310587  .1) | Forward GGGAGTCTAGATCCTTGGACCTCTTG CTGGAT  Reverse GGGAGGGCCGGCCCTGTCTCCCATCC CCTTTCC | 148 |
| --- | --- | --- |
| Mef2d 3′-UTR 5140-5276 (NM_001310587  .1) | Forward GGGAGTCTAGAAAGAGGGAAGAAGG GAGGGA  Reverse GGGAGGGCCGGCCGAAAGAAATAAT GAAAACAAACCAACAGC | 158 |
| Snrnp200 CDS 3037-3114, ex21-22 (NM_177214.5) | Forward GGGAGAAGCTTAGCCACGATGGGCA ACTTCCAGGTGACAGAAC  Reverse  GGGAGTTCCATGGTGTTGTAGGTCTG CACAGTATCATTGGT | 113 |

**Supplementary Table S2.** Results of the search for Plaur-miR1-5p target genes using the DianaTools webservice*.*

| **Position** | **Ensembl transcript ID** | **Binding score** | **Loci** |
| --- | --- | --- | --- |
| 1 | **ENSMUST00000174353 (*Emx2*)** | 0.997770 | 3′-UTR |
| 2 | ENSMUST00000099981(*Ttn*) | 0.996939 | CDS |
| 3 | ENSMUST00000088419(*Mbnl2*) | 0.993618 | 3′-UTR |
| 4 | ENSMUST00000187455(*Gm28360*) | 0.990815 | 3′-UTR |
| 5 | ENSMUST00000056006(*Onecut1*) | 0.988168 | 3′-UTR, CDS |
| 6 | ENSMUST00000175965(*Onecut2*) | 0.984653 | 3′-UTR, CDS |
| 7 | ENSMUST00000048702(*Papd4*) | 0.979442 | 3′-UTR |
| 8 | ENSMUST00000095767(*Etv1*) | 0.978688 | 3′-UTR |
| 9 | ENSMUST00000174734(*Kmt2c*) | 0.968095 | 3′-UTR |
| 10 | ENSMUST00000043285(*Gm11992*) | 0.966855 | 3′-UTR |
| 11 | ENSMUST00000066187(*Nhlh2*) | 0.965869 | 3′-UTR |
| 12 | ENSMUST00000111416(*Ildr2*) | 0.964307 | 3′-UTR, CDS |
| 13 | ENSMUST00000078944(*Phf6*) | 0.962477 | 3′-UTR |
| 14 | ENSMUST00000029588(*Larp7*) | 0.960559 | 3′-UTR, CDS |
| 15 | ENSMUST00000119972(*Dnajb3*) | 0.954241 | 3′-UTR, CDS |
| 16 | ENSMUST00000068714(*Sos1*) | 0.948827 | 3′-UTR, CDS |
| 17 | ENSMUST00000030134(*Rad23b*) | 0.946043 | 3′-UTR |
| 18 | ENSMUST00000034349(*Nae1*) | 0.940100 | 3′-UTR, CDS |
| 19 | ENSMUST00000159690(*Arrdc3*) | 0.938964 | 3′-UTR |
| 20 | ENSMUST00000023101(*Slc38a4*) | 0.938064 | 3′-UTR, CDS |

3′-UTR, 3′ untranslated region; CDS, coding DNA sequence.

**Supplementary Table S3.** Results of the search for Plaur-miR1-3p target genes using the DianaTools webservice.

| **Position** | **Ensembl transcript ID** | **Bindingscore** | **Loci** |
| --- | --- | --- | --- |
| 1 | **ENSMUST00000001455 (Mef2d)** | 0.989700 | 3′-UTR |
| 2 | ENSMUST00000005860(Pvalb) | 0.979949 | 3′-UTR |
| 3 | ENSMUST00000078944(Phf6) | 0.972257 | 3′-UTR |
| 4 | ENSMUST00000198931(Cdkl5) | 0.949611 | 3′-UTR |
| 5 | ENSMUST00000080132(4921509C19Rik) | 0.946798 | 3′-UTR |
| 6 | ENSMUST00000167085(Spats2l) | 0.940340 | 3′-UTR, CDS |
| 7 | ENSMUST00000109586(Sertad2) | 0.938621 | 3′-UTR |
| 8 | ENSMUST00000042779(Zbtb1) | 0.928436 | 3′-UTR, CDS |
| 9 | ENSMUST00000026093(Btbd1) | 0.919734 | 3′-UTR |
| 10 | ENSMUST00000019878(Leng1) | 0.917817 | 3′-UTR, CDS |
| 11 | ENSMUST00000123418(Ank1) | 0.913491 | 3′-UTR |
| 12 | ENSMUST00000105362(Dazap1) | 0.912172 | 3′-UTR, CDS |
| 13 | ENSMUST00000029141(Mmp24) | 0.903432 | 3′-UTR, CDS |
| 14 | ENSMUST00000109913(Nr3c2) | 0.900063 | 3′-UTR |
| 15 | ENSMUST00000199256(Ldb2) | 0.898156 | 3′-UTR |
| 16 | ENSMUST00000001548(Itga3) | 0.897849 | 3′-UTR, CDS |
| 17 | ENSMUST00000164960(Rasgef1a) | 0.897492 | 3′-UTR |
| 18 | ENSMUST00000133654(Acss2) | 0.895093 | 3′-UTR |
| 19 | ENSMUST00000182746(Gm26920) | 0.894848 | 3′-UTR |
| 20 | ENSMUST00000107166(Tenm4) | 0.891823 | 3′-UTR, CDS |

3′-UTR, 3′ untranslated region; CDS, coding DNA sequence.

# REFERENCES:

- 1. Kanellos I., Vergoulis T., Sacharidis D., Dalamagas T., Hatzigeorgiou AG, Sartzetakis S., Sellis T. (2014) MR-microT: a MapReduce-based MicroRNA target prediction method. *SSDBM 2014*: **47**, doi: 10.1145/2618243.2618289
  2. Reczko M., Maragkakis M., Alexiou P., Grosse I., Hatzigeorgiou A.G. (2012) Functional microRNA targets in protein coding sequences. *Bioinformatics,* **28(6)**, doi: 10.1093/bioinformatics/bts043
  3. Semina, E., Rubina, K., Sysoeva, V., Rysenkova, K., Klimovich, P., Plekhanova, O. and Tkachuk, V. (2016) Urokinase and urokinase receptor participate in regulation of neuronal migration, axon growth and branching. *Eur. J. Cell Biol.*, **95**, 295– 310.10.1016/j.ejcb.2016.05.003
